# Supplementary material for: Eocene Loranthaceae pollen pushes back divergence ages for major splits in the family
Source: PeerJ. 2017 Jun 7;5:e3373. doi: 10.7717/peerj.3373 (PMC5466002; doi:10.7717/peerj.3373)

NCBI GenBank Harvest, 4/3/2014

This is a partially literal copy of File S2 in the online supplementary material to Grímsson et al. (2016). Emendations/modifications are highlighted by blue font.

Search strings taxa and gene regions

Taxa

Loranthaceae: txid3963[Organism:exp]

Gene regions

*mat*K gene: … AND matK [gene]

*rbc*L gene[1]: … AND rbcL [gene] NOT "atpB-rbcL" [title]

trnLLF region (*trn*L intron, *trn*L-*trn*F spacer): … AND "trnL-trnF" [title][2]

18S rDNA: … AND (18S [title] or "small subunit" [title]) NOT "internal" [title]

ITS region of the 35S rDNA[3]: … AND "5.8S" [title]

25S rDNA: … AND 26S [title] NOT "internal" [title]

[1] Four sequences of *Benthamina* sp. SH-2010 (Tobe et al., unpubl.; AB586377–AB586380) not caught by search and were manually added.

[2] The covered region in these sequences includes also most of the upstream *trn*L intron

[3] One ITS2 accession (*Tristerix penduliflorus*, DQ442975, Amico et al., *Am. J. Bot.*, 2007) comprising 197 bp (ITS2 only) will not be caught. Being only a short fragment without extra information, the sequence was not manually added.

Processing

Gene bank flatfiles were transferred into FASTA-format using GBK2FAS (Göker et al. 2009, see batch file for options). Auto-alignments were generated with MAFFT v. 6.935b (Katoh et al. 2005; Katoh & Standley 2013), optimal algorithm chosen by the programme.

Dataset	Algorithm
[1] 18S	L-INS-i
[2] ITS	FFT-NS-i
[3] 25S	FFT-NS-i
[4] *mat*K	L-INS-i
[5] trnLLF	FFT-NS-i
[6] rbcL	L-INS-i

All alignments were inspected by eye, and their ends were trimmed. As a rule, we truncated the alignments for less than four accessions at the 5’ and 3’ ends.

Nuclear-encoded 18S rDNA (Fig. S1-1)

General—The data comprise near-complete 18S rDNA sequences showing only restricted length-polymorphism at pos. 184–189 (CT-dom. motif), 223–233 (upstream-GC, downstream CT) and within the terminal loop of stem 49 (tl49) at the 3’ end of the 18S rDNA. Several single-nucleotide (nt) gaps are introduced in which a single of the sequences shows an extra nucleotide typically the same then before or after the gap.

Curation—Isolated single-nucleotide gaps are likely due to sequencing/editing artefact rather than representing actual mutations and have been eliminated from the alignment. Right-aligned the length-polymorphic motives at pos. 184ff; left-aligned pos. 229ff; centre-aligned tl49-motives. Several accessions (*Gaiadendron punctatum*, L24143; *Helicanthes elastica*, EU544328; *Erianthemum dregei*, L25679; *Oedina pendens*, EU544336; *Ligaria cuneifolia*, L24152; *Struthanthus oerstedii*, L24421) show increased amount of deviations within strongly conserved stem regions (pseudogeny?). Where conclusive comparative data was lacking, the sequence was kept as-is, otherwise blanked out (replaced by missing data symbol).

Problematic data—Accession AF039074 (*Decaisnina hollrungii*, no voucher information included; Elytrantheae) differs from the sequence of its congener, hence, the misplacement of *Decaisnina* in the 18S tree based on the reduced data set.

Nuclear-encoded ITS region

General—The nrDNA spacers are highly divergent and length-polymorphic at the family level, resulting in a very gappy auto-alignment. The ITS1 cleavage site region, the 5.8S rDNA, and the generally more conserved parts of the ITS2 are successfully recognised as alignable blocks due to their high conservation level and overall lack of length-polymorphism.

Note: The data was not included in family-wide analyses due to potential alignment artefacts on the overall topology.

Curation—Except for right-aligning the start of the 5.8S rDNA, the alignment was left un-changed.

Problematic data—Three accessions show likely pseudogenous mutations in the 5.8S rDNA (*Dendrophthoe constricta*, DQ333840, 3’ part; *Helixanthera parasitica*, DQ333823, central part; *Psittacanthus schiedeanus*, DQ333859, 3’ part, 5’ part with small missing data blocks).

In case of DQ333840 mutational patterns linked to pseudogeny are also apparent in the ITS1 and start of ITS2 (identifiable by comparison with the second accession of the genus and other members of the Lorantheae).

In the case of DQ333823, pseudogeny is less obvious in the ITS1 and ITS2: the sequence appears to be a mix of ITS variants with different levels of pseudogeny as indicated by polymorphic calls (Y and R at overall conserved C and G positions).

DQ333859 may be a chimera (or recombinant) of a non-pseudogenous and (weakly) pseudogenous ITS variants: the area of overlap between typical forward and reverse reads has been blanked out by the authors indicating problems with the direct PCR sequencing. Lacking comparative data of this genus/species, the sequence was kept as-is.

The 3' portion of the ITS2 in *Notanthera heterophylla* DQ333855 is entirely different from other accessions of the subtribe/tribe and markedly off-alignment. The strand portion finds no similar hits in gene banks. Based on the general distinctness of the ITS of this taxon, the 3' ITS2 may be genuine and has been kept as-is.

One of the two accessions of *Taxillus* (*T. chinensis*, JX177497) differs strongly from the other sequence and is essentially off-alignment within ITS1 and the larger part of ITS2. Subsequent MEGABLAST identifies the sequence as *Viscum* (same order, family Viscaceae).

Nuclear-encoded 25S rDNA data (Fig. S1-2)

General—Data comprise near-complete 25S rDNA sequences. Variation and minor length-polymorphism is limited to the D1–D4 regions of the 25S rDNA. Markedly divergent patterns in generally sequential- and structural-conserved regions are limited to a few accessions (→ **Problematic data**)

Curation—Left-aligned C-rich motif at pos. 2078ff.

Problematic data—Accessions of *Diplatia furcata* (EU544368), *Helicanthes elastica* (EU544375), *Lepidaria* cf. *forbesii* (EU544378), *Ixocactus sp.* (EU5443779), *Spraguenella rhamnifolia* (EU544401), *Loxanthera speciosa* (EU544382) show an increased number of substitutions in generally (clade or family) conserved regions. In the case of *Ixocactus* the sequence inflicts single-nt gaps.

In case EU544368 many deviations are indicative for pseudogeny (G→A, C→T) in addition to deviations that may be linked to bad sequence reads/editing artefacts (e.g. AATTA at pos. 485–489 instead of the consensual ATTGA). The sequence was excluded from analysis.

EU544375 (*Helicanthes elastic*; Nickrent 4906) represents a unique 25S rDNA fragment, with best BLAST hits of 92–93% identity covering a wide range of taxa and clades (Solanales: *Schizanthus*, Apiales: *Bolax*; Saxifragales: *Saxifraga, Telesonix*; Ranuculales: *Borax*; etc.); top hits did not include members of Loranthaceae. The sequence was tentatively excluded from analyses as putative contaminant. The 18S sequence from the same voucher is equally problematic.

Accession EU544378 (Nickrent 4044, labelled as *Lepidaria* cf. *forbesii*) shows increasing amount of pseudogenous positions (G→A, C→T mutations in generally conserved regions) towards the 3' part. The sequence was excluded from analysis as showing tendency towards pseudogeny.

The same, but to a lesser degree applies to accessions EU544368 (*Diplatia furcata,* Nickrent 2824) and EU544382 (*Loxanthera speciosa,* Nickrent 4026). The latter was tentatively kept for analysis due to the importance of the taxon for the phylogenetic framework.

Accession EU5443779 (Nickrent 4332, labelled as *Ixocactus* sp. [= *Phthirusa*]) is composed of two strands. The first strand (170 nt from the 5' end) finds best BLAST-hits among the Loranthaceae, but lower identity than usually found in when BLASTing a member of the family. Then comes a sequencing gap and the following remaining c. 1200 nt find highest identities (>90%; obtained with MEGABLAST) only with members of other families of the Santalales (*Osyris,* Santalaceae; *Haloragis*, Halogaraceae) and unrelated singletons such as *Eucnide bartonioides* (JF321124), *Loasa vulcanica* (JF321125; Cornales), *Fendlera rupicola* (AY260041), *Dryas octopetala* (JF317384; Rosales), and *Eucryphia lucida* (AF036494, Oxalidales). Based on this result it is uncertain whether the sequence is genuine or a mislabelling/artificial chimera; hence, it was tentatively excluded from all analyses.

Accession EU544401 differs from the general sequences types within members of its clade and Loranthaceae in general by potential pseudogene-like mutations in addition to sequence deviations that may be linked to sequencing/editing artefacts. Because of its overall dissimilarity to other Loranthaceae sequences in family diagnostic regions, MEGABLAST (performed 9/2/2016) failed to retrieve any Loranthaceae 25S within the listed hits. Note that the pollen of one species formerly included in *Ixocactus* (*Phthirusa hutchisonii*) does not confirm with morphotypes typical for Loranthaceae (Grímsson, Grimm & Zetter 2017)

Plastid *mat*K gene (Fig. S1-3)

General—Data includes up-to-near-complete *mat*K sequences starting with the first codon in three accessions (stop codon seems to be not covered in any accession). More than 3-taxon coverage starts at the 27th codon (alignment was accordingly truncated). Length-polymorphism is restricted to singleton duplications or eliminations.

Curation—In regions with duplications/eliminations, the general alignment was adapted for codon positions when necessary. Defect (due to missing single-nucleotides) and incomplete codons were blanked (replaced by missing data symbol, "?").

Problematic data—The 5' part of accession EU544409 (*Aetanthus nodosus*) is markedly different from other members of its tribe and the entire family. MEGABLAST revealed that the first c. 260 nt are highly identical to accession of several species of *Acacia* (Fabales). The remainder of the sequence falls within the variation of the Loranthaceae. Thus, the accession is a (artificial) chimera of contaminated material and unverified *Aetanthus* (no comparative data available of the genus), and has to be excluded from analysis.

Two accessions, EU544414 (*Baratranthus axanthus,* Nickrent 4029) and EU544435 (*Lepeostegeres lancifolia,* Nickrent 4041) are placed within wrong subtrees (cf. Nickrent et al. 2010). In case of *Baratranthus,* other gene regions (18S, 25S, trnLF) sequenced from the same voucher are unsuspicious.

Plastid trnLLF region (Fig. S1-4)

General—The data includes the *trn*L intron, the 3’ *trn*L exon (identified by comparison to *Panax ginseng* complete plastome, KC686331), the *trn*L-*trn*F intergenic spacer and up to nearly complete *trn*F genes (15–21 nt missing in longest sequences). Most length-polymorphism in the intron is linked to duplications in a single or few sequences and a prominent multiple-A motif (pos. 190ff)

Curating—Alignment in singletons at pos 132–166 (in three accessions) were corrected for duplication patterns. Left-aligned pos. 252–266, and 633–648. Local alignments in the *trn*L-*trn*F spacer appear to be suboptimal but were kept as-is.

Odd placements—The trnLLF region is the only gene region included in the final dataset, where genera (to some degree: species) are usually represented by more than a single accession. The single-gene tree reveals several issues with the currently available sequence data:

Psittacanthinae clade: The putative sister genera *Cladocolea* and *Strutanthus* (Vidal-Russell & Nickrent 2008; Su et al. 2015; Grímsson, Grimm & Zetter 2017) are not resolved as distinct clades, despite a general relatively high branch support in the corresponding subtree. The only representative of genus *Phthirusa*, *P. inorna* (former *Ixocactus inornus*) is nested within the *Tripodanthus* subclade (genus’ root poorly supported) comprising multiple accessions for two of the three species in the genus. Most conspicuously, the accessions representing the putative sister genera *Oryctanthus* (*O. occidentalis*) and *Passovia* (*P. pyrifolia*) of Wilson & Calvin (2006) and Vidal-Russell & Nickrent (2008) are mixed up. A shorter branched subclade comprises accession DQ340608 of *P. pyrifolia* (Calvin & Wilson CR01-3) and EU544501 of *O. occidentalis* (Nickrent 2763), whereas the other is remarkably long-branched and includes the respective counterparts (DQ340613, *O. occidentalis*, Calvin & Wilson CR01-11; EU544504, *P. pyrifolia*, Nickrent 2762). Comparative chloroplast data are only available for the Nickrent 2762 and 2763 vouchers; in the *mat*K tree, *P. pyrifolia* is long-branched whereas *O. occidentalis* is shorter branched. Thus, we assume that the Calvin and Wilson sequences have been mixed-up during upload.

Others: Visible distinct accessions separate the two *Lepidaria* samples (*L. oviceps*, DQ340602, Calvin & Wilson B02-19; *L.* cf. *forbesii*, EU544492, Nickrent 4044) and the three covered *Amyema* species. In the latter case, *mat*K data are available for comparison and confirming the ‘non-monophyly’ of the genus. Accession DQ340578 (*Taxillus wiensii*; A. Robertson 7364) places in the wrong subtree, as sister to *Vanwykia*. The Scurrulinae *Scurrula* and *Taxillus* group in a distinct subclade in all single-gene trees. Thus, we have to assume that accession DQ340578 is either mislabelled, or that the according species is not a member of the Scurrulinae.

Plastid *rbc*L gene (Fig. S1-5)

General—The matrix covers the 469 codons of the *rbc*L gene (starting with the 8th codon, last three codons of complete *rbc*L not represented in GenBank data; reference sequence *Panax ginseng* KC686331). Most variable sites (~75%) refer to the 3rd codon position.

Curating—Alignment of *rbc*L is straightforward (generally no length polymorphism). The region is generally conserved at the genus/subtribe level, which facilitates the identification of putative mislabelled sequences. The data includes one unidentified Loranthacea *rbc*L, which was not considered for further analyses.

Problematic data— Three sequences of *Taxillus chinensis* (JF949992; JN687568; KF447376) differ markedly from the other eight sequences of the species and other members of the genus/tribe (visible from pos. 250 onwards). MEGABLAST identified JF949992 as Elytrantheae/Gaiadendreae (99% identity; verified on the alignment) and JN687568 as Viscaceae, another family of the Santalales (most similar to *Viscum*, 97% identity; no Loranthaceae among the top hits). The sequences either represent mislabelled or misidentified specimens. KF447376 received no significant hit with identity >93% (best hits with *Scurulla,* the sister genus of *Taxillus*, generic affinity verified at hand of the full alignment). The sequence represents either a bad sequence read or a strongly aberrant *rbc*L gene (potential pseudogene). All three sequences were excluded from analyses.

A similar sequence type than in JN687568 is found in the only accession of *Oryctanthus cordifolius* (JQ592409), differing from the otherwise near-identical sequence of Psittacanthinae. MEGABLAST identifies the sequence accordingly as Viscaceae *rbc*L (*Phoradendron*; 98% identity). The sequence is either mislabelled or *Oryctanthus* is not a Loranthaceae and has been omitted.

Another problematic sequence is the one representing *Macrosolen brandisianus* (JN687566) which differs increasingly towards the 3' end from other accessions of the genus/tribe. Deviation is usually related to same-nucleotide repeats (e.g. G**GT**GGT instead G**TG**GGT in all other Lorantheae at pos. 349ff; AA**T**TTG instead AA**A**TTG as pos. 460ff). The problematic sequence part encompasses 50% of the total sequenced strand (c. 500 nt), the first half of the sequence is identical to other Lorantheae. The sequence was tentatively excluded from analyses.

Odd placements—The *rbc*L data includes genuine sequences from phylogenetic studies as well as short(er) sequences from barcoding studies. The latter are often problematic, mislabelled accessions are not uncommon (see *Problematic data*). Moreover, and contrasting statements in many barcoding papers, there is so far no conclusive evidence that *rbc*L can be used as barcode at low hierarchical levels in plants. Reported high species-identification levels with *rbc*L fragments in barcoding studies are simply due to incomprehensive species sampling of large genera (G. Grimm, pers. obs.) The currently available *rbc*L data on Loranthaceae supports this observation: the resolution of the *rbc*L tree is generally poor. Identical sequences can be shared by species of the same genus, sometimes between genera. Based on the current, relatively taxon-limited data it is not possible to distinguish between potentially representative vs. mislabelled accession in critical cases (*Dendrophthoe, Helixanthera, Struthanthus, Taxillus*). With respect to this taxonomic uncertainty, the generally weak signal and the limited species coverage (23 species with genuine *rbc*L data compared to 44/45 covered by *mat*K and trnLF) of the *rbc*L data, we excluded this gene region from further analyses.

Set-up for phylogenetic analyses and dating

Phylogenetic tree inferences and branch support analyses relied on maximum likelihood (ML) as the optimality criterion, branch support was estimated using non-parametric bootstrapping. All analyses, files can be found in subfolder *ML* in OSA, were done using RAxML v.8.2.4 (Stamatakis 2014). RAxML was invoked using custom shell files (included in subsequent subfolders in the OSA). All inferences used the -f a option that performs a (in our case, fast; option -x) bootstrapping analyses with subsequent inference of the 'best-known' ML tree under a general-time-reversible substitution model allowing for site rate variation, modelled using a gamma distribution (GTR+Γ model) in the final optimisation step. During bootstrapping and the first (fast) tree inference step, the per-site approximation for the gamma distribution was used (option -GTRCAT; Stamatakis 2006). Number of necessary bootstrap replicates were determined by the extended majority rule consensus criterion (Pattengale et al. 2009), bootstrap analyses were automatically cut off when reaching a maximum of 1000 replicates.

We use a seven-step protocol

***Step 1:* Preanalysis—**Using the initial alignments (not consensed, not concatenated gene bank accessions), we inferred single-gene trees for five of the six harvested regions: 18S, 25S, *mat*K, trnLLF, *rbc*L. This step allowed us to further check for problematic accessions not directly visible or overlooked during the visual inspection of the alignments and to assess the general level of discriminative signal in the single gene regions (see **Processing**).

***Step 2:* Consensing—**Modal and strict species-consensus sequences were computed from the initial alignments using the programme G2CEF (Göker & Grimm 2008); gaps were treated as missing data or fifth state (batch and files are included in the subfolder *Step2_Consensi* in this Online Supplementary Archive, OSA). For further analyses the strict consensus sequences with gaps treated as missing were used.

***Step 3:* Inference of comprehensive species trees—**Using the species-consensus alignments, we produced three concatenated matrices: (1) including all four gene regions (18S, 25S, *mat*K, trnLLF), (2) a nuclear-only matrix including 18S and 25S rDNA, and (3) a plastid-only matrix including the *matK* gene and the trnLLF region. ML analyses were run partitioned and unpartitioned, including or excluding the relatively gappy, length-polymorphic *trn*L-*trn*F spacer (i.e. analyses used five matrices with different gene coverage). The partitioning scheme was as follows:

- 18S rDNA and 25S rDNA treated as independent partitions
- First, second and third codon position of *mat*K gene treated as independent partitions
- Intron and intergenic spacer treated as independents partitions, exons (*trn*L 3' exon, *trn*F gene) included in the same partition.

***Step 4:* Clock rooting—**Data sets including selected outgroups (Wilson & Calvin 2006; Vidal-Russell & Nickrent 2008) or representatives of all/most Santalales families (e.g. Su et al. 2015; see files provided in the OSA to Grímsson, Grimm & Zetter 2017) failed to produce unambiguous support for

the exact position of the Loranthaceae root, although trees based on combined data usually will recognise *Nuytsia* as the first diverging branch in the Loranthaceae. All potential outgroups are (extremely) long-branched and *Nuytsia* is one of the most distinct Loranthaceae. Thus, ingroup-outgroup long-branch attraction may be inevitable (as detailed by GWG in Grímsson, Grimm & Zetter 2017, file S6). Hence, a clock-rooting (cf. Renner et al. 2008), was tried using the five matrices from step three. For each of the matrices we performed a BEAST (v. 1.8.2; Drummond & Rambaut 2007; Drummond et al. 2012) run under partition specific substitution models, unconstrained tree topology, a Yule tree prior and uncorrelated log-normal clock prior [ucld.mean ~Gamma(0.001, 1000)]. The best fitting substitution models per partition, among the available in BEAST, were selected with JMODELTEST (Darriba et al. 2012). The selected model for most partitions (rDNAs, *mat*K codon positions, *trn*L-*trn*F spacer) was GTR+Γ, whereas HKY+Γ was suggested for *trn*L intron and HKY+I for the tRNA. Each BEAST analysis was conducted for $2*10^7$ generations with a sampling frequency of 0.001. To explore the effect of missing data, we performed the clock-rooting also with the reduced dataset of 42 species covering the four best-sampled and alignable gene regions (18S, 25S, *mat*K and trnLLF) that was used for the final dating step (***Step 6***). The *rbc*L gene was excluded since it was represented only by a minority of the samples. The analysis was run under the same priors as before.

Step 5: **Inference of preliminary dated phylogenies including all data—**Using the two all-comprehensive matrix from Steps 3 and 4, i.e. the matrix including all four gene regions (18S, 25S, *mat*K, trnLLF), dated phylogenies were inferred for three different rooting scenarios: traditional (following earlier studies) outgroup-based root as informed by *Nuytsia*, the same root is found via clock-rooting of the reduced 42-taxon, 4-gene region dataset; the clock-informed root based on the all-taxon datasets recognising two main clades: Lorantheae vs. Psittacantheae+root parasites; and a pollen-type-informed root recognising *Tupeia* as sister to all other Loranthaceae (cf. Grímsson, Grimm & Zetter 2017). Table S1-1 lists the used age constraints including short discussions (see also subsections *Use as age constraint* included in the pollen descriptions in the main-text). Dating was performed with BEAST v. 1.8.2 (Drummond & Rambaut 2007; Drummond et al. 2012). Bayesian optimisation used four data partitions with the following nucleotide substitution models: GTR+I for 18S and GTR+Γ for 25S, *mat*K, and trnLLF, respectively. The Monte Carlo Marko chains (MCMC) were run twice for $5*10^7$ million generations under an uncorrelated lognormal clock model. All age calibration priors were modelled as normal distributions around the midpoint of the known time intervals. For rooting scenario 2, the analysis of the comprehensive matrix did not converge after the set number of generations, hence, no result tree is included in the according subfolder. For the other two rooting scenarios, the high gappyness of the comprehensive matrix caused similar problems (relatively low ESS for several parameters, late convergence of runs). For this reason, the final dating analysis used a reduced species set, only including those members of each lineage that were best-covered by data for all four gene regions.

Table S1-1

MRCA	Rooting scenario (r.sc.) 1		Rooting scenario 2		Rooting scenario 3		Scenario 4: 'true tree' (Su et al., 2015) enforced	
	**Fossil**	**Age**	**Fossil**	**Age**	**Fossil**	**Age**	**Fossil**	**Age**
All Loranthaceae	Miller Clay Pit MT1	47.8–41.2	Stolzenbach MT	47.8–41.2	(not constrained)		Same as for r.sc. 1/2	
All Loranthaceae except *Nuytsia*	(not fixed)[a]		(incompatible with tree)		(incompatible with tree)		(not fixed)[a]	
All Loranthaceae except *Tupeia*	(incompatible with tree)		(incompatible with tree)		Miller Clay Pit MT1(–3), Stolzenbach MT	47.8–41.2	(incompatible with tree)	
Elytrantheae + Lorantheae	(incompatible with tree)		(incompatible with tree)		(incompatible with tree)		Profen MT2–5	41.2–38
	(incompatible with tree)		(incompatible with tree)		(incompatible with tree)			
Lorantheae	Changchang MT	47.8–37.8	Same as for r.sc.1		Same as for r.sc.1		Same as for r.sc. 1	
Psittacantheae + root parasites	(incompatible with tree)		Miller Clay Pit MT1	47.8–41.2	(incompatible with tree)		(incompatible with tree)	
Notanthera + Elytrantheae	Profen MT2–5	41.2–38	Same as for r.sc.1		Same as for r.sc.1		(incompatible with tree)	
Psittacanthinae[c]	(Miller Clay Pit MT2, 3) Aamaruutissaa MT	(47.8–41.2) 42–40	Same as for r.sc.1		Same as for r.sc.1		(not fixed)[d]	
Notanthera + Psittacanthinae	(incompatible with tree)		(incompatible with tree)		(incompatible with tree)		Aamaruutissaa MT[d]	42–40

[a] Could be constrained to the same minimum age than MRCA of all Loranthaceae (co-eval: Miller Clay Pit MT2-MT3 → Psittancanthinae, and Stolzenbach MT → Lorantheae)

[b] The Theiss MT represents a putative ancestral, primitive (plesiomorphic) pollen of the Lorantheae lineage, less derived than the Changchang MT. Being younger but linked to a deeper node (formation of Lorantheae vs. radiation of core Lorantheae, Clade I/J according Vidal-Russell & Nickrent), it has little use as additional minimum age prior.

[c] All pollen addressed as Miller Clay Pit MT2 and MT3 and Aamaruutissaa MT are very similar to that of two species of modern *Tripodanthus* and could be used to constrain the divergence of the *Tripodanthus* lineage from the remainder of the Psittacanthinae. Nevertheless, they are used as minimum age priors for a deeper node: the MRCA of all Psittacanthinae. This was done to compensate for missing information (pollen type of *Phthirusa inorna*, the potentially first branching modern Psittacanthinae is unknown; *Tripodanthus* pollen may represent a primitive type within the lineage) and with respect to ambiguous signal regarding deep relationships in the Psittacanthinae subtree.

[d] Although the Miller Clay Pit MT2 and MT3 are possibly older, we opted to use the more precisely dated Aamaruutissaa MT as age constraint, placed one node up in the other trees constraining for the root age of the Psittacanthinae. This may result in underestimated (slightly too young) ages (see *Results* in the main text), but avoids having to use similar age constraints for two nodes in a line (i.e. the MRCA of *Notanthera* and Psittacanthinae and the root node of Psittacanthinae).

***Step 6:* Final inference of dated phylogenies—**To compensate for eventual missing data artefacts on the estimates, we performed the same set of analyses with a taxon-reduced data set, a data set that only included 42 species with data for all five gene regions. The general set-up (priors etc.) was the same as for the preceding step, further details can be extracted from the xml-files provided in the according subfolder in the OSA.

At this step, we performed an additional analysis (Scenario 4, Table S1-1) with a partly constrained backbone phylogeny. During the review of Grímsson, Grimm & Zetter (2017), one anonymous reviewer, an expert on plant parasites in general and Loranthaceae in particular, informed us about the "correct" placement of several taxa (essentially the topology seen in Su et al. 2015, fig. 1B; but see figs S6 and S7 in the supplement to that paper). The topology of the "correct tree" differs in several aspects from the topologies that can be inferred from the here used data set(s). The data basis regarding the covered (coding) genes was similar but not identical in both studies, the additionally used genes (*RPB2, accD, mat*R) in Su et al. (2015) cover very few of the Loranthaceae and mainly (*RPB2, mat*R) enforce the placement of the Mystropetalaceae as sister of Loranthaceae. At the same time these additional gene regions inflict *mat*K-conflicting signals (Grímsson, Grimm & Zetter 2017, file S6), the latter being the gene region that informs most splits seen in the Su et al. (2015) tree. Our dataset includes instead the non-coding trnLLF region, which has a broader taxon coverage and a much higher divergence than the coding regions included in Su et al. Our dataset also lacks distant, (extremely) long-branching outgroups (there is e.g. no plastid data available for the Mystropetalaceae). Furthermore, we use species-consensus sequences instead of selected single individuals used as placeholder, thus, our matrix has a lower gappyness. This may affect directly the optimised topology because the signal from individual gene regions is apparently not fully compatible in the Loranthaceae (see Grímsson et al. 2016, file S1). To compensate for the incapability of our data set to infer "correct" relationships, we hence did an additional dating analysis in which we constrained the topology according to that reviewer's views. The three root parasites were constrained to form a grade: following up from the most distinct root parasite and sister to the remainder of the Loranthaceae (*Nuytsia*), come the relatively long-branched *Atkinsonia* and the short-branched *Gaiadendron*. Within the aerial parasite clade, most New World taxa are collected within an according clade. In this 'New World' clade, the putative sister taxa *Ligaria* and *Tristerix* (Ligarinae) are constrained as sister to *Notanthera* and the Psittacanthinae. The conservative pinning of Elytrantheae fossils found at Profen requires the identification of the sister group of the Elytrantheae (which is *Notanthera* in all optimised topologies, apparently a "wrong" placement). Following the topology of Su et al. (2015), we constrained the Elytrantheae as sister to the Lorantheae.

***Step 7:* Post-analyses test (comparison of Bayes factors)**

For each of the four topological configurations we estimated the marginal likelihood with two approaches, stepping-stone and path-sampling, both of which are implemented in BEAST (Baele et al. 2012; Baele et al. 2013), with 100 path steps with $20*10^7$ chain length for each. For selecting the rooting

scenario that best fit our data, we compared the marginal likelihood estimate of each run using Bayes factors (Kass & Raftery 1995).

Documentation

All analysis files are included in the OSA in corresponding subfolders. See ReadMe.txt in top folder for file labels and brief description of content. The OSA is mirrored at www.palaeogrimm.org/data; if you have questions regarding the data, files, and inferences contact GWG (mail-to: loranths@palaeogrimm.org).

Figures (appended at the end of the file)

Single-gene trees based on the harvested, unconsensed/-concatenated sequence data and optimised under ML; visibly problematic – as directly deduced from the alignment – sequences not included. Branches, subtrees, etc. referring to major groups (tribes/subtribes) of Loranthaceae are coloured accordingly; branch thickness indicates bootstrap support under ML (see in-figure legends). Highlighted by red font and in bold, mislabelled accessions detected via the tree inference and excluded from subsequent analyses; accessions highlighted by orange font (bold) may be problematic but tentatively kept due to the unavailability of comparative data.

References

Baele G, Lemey P, Bedford T, Rambaut A, Suchard MA, Alekseyenko AV. 2012. xxx. *Molecular Biology and Evolution* 29:2157–2167.

Baele G, Li WLS, Drummond AJ, Suchard MA, Lemey P. 2013. xxx. *Molecular Biology and Evolution* 30:239–243.

Darriba D, Taboada GL, Doallo R, Posada D. 2012. jModelTest 2: more models, new heuristics and parallel computing. *Nature Methods* 9:772–772.

Drummond AJ, Rambaut A. 2007. BEAST: Bayesian evolutionary analysis by sampling trees. *BMC Evolutionary Biology* 7:214.

Drummond AJ, Suchard MA, Xie D, Rambaut A. 2012. Bayesian phylogenetics with BEAUti and the BEAST 1.7. *Molecular Biology and Evolution* 29:1969–1973.

Göker M, García-Blázquez G, Voglmayr H, Tellería MT, Martín MP. 2009. Molecular taxonomy of phytopathogenic fungi: a case study in *Peronospora*. *PLoS ONE* 4:e6319.

Göker M, Grimm GW. 2008. General functions to transform associate data to host data, and their use in phylogenetic inference from sequences with intra-individual variability. *BMC Evolutionary Biology* 8:86.

Grímsson F, Grimm GW, Zetter R. 2017. Evolution of pollen morphology in Loranthaceae. *Grana* DOI:10.1080/00173134.2016.1261939.

Kass RE, Raftery AE. 1995. Bayes factors. *Journal of the American Statistical Association* 90:773–795.

Katoh K, Kuma K, Toh H, Miyata T. 2005. MAFFT version 5: improvement in accuracy of multiple sequence alignment. *Nucleic Acids Research* 33:511–518.

Katoh K, Standley DM. 2013. MAFFT multiple sequence alignment software version 7: improvements in performance and usability. *Molecular Biology and Evolution* 30:772–780.

Pattengale ND, Masoud A, Bininda-Emonds ORP, Moret BME, Stamatakis A. 2009. How many bootstrap replicates are necessary? In: Batzoglou S, ed. *RECOMB 2009*. Berlin, Heidelberg: Springer-Verlag, 184–200.

Renner SS, Grimm GW, Schneeweiss GM, Stuessy TF, Ricklefs RE. 2008. Rooting and dating maples (*Acer*) with an uncorrelated-rates molecular clock: Implications for North American/Asian disjunctions. *Systematic Biology* 57:795-808.

Stamatakis A. 2006. Phylogenetic models of rate heterogeneity: A high performance computing perspective. Proceedings of 20th IEEE/ACM International Parallel and Distributed Processing Symposium (IPDPS2006), High Performance Computational Biology Workshop. Rhodos, Greece, April 2006. p [on CD, no page nos].

Stamatakis A. 2014. RAxML version 8: a tool for phylogenetic analysis and post-analysis of large phylogenies. *Bioinformatics* 30:1312–1313.

Su H-J, Hu J-M, Anderson FE, Der JP, Nickrent DL. 2015. Phylogenetic relationships of Santalales with insights into the origins of holoparasitic Balanophoraceae. *Taxon* 64:491–506.

Vidal-Russell R, Nickrent DL. 2008. Evolutionary relationships in the showy mistletoe family (Loranthaceae). *American Journal of Botany* 95:1015–1029.

Wilson CA, Calvin CL. 2006. An origin of aerial branch parasitism in the mistletoe family, Loranthaceae. *American Journal of Botany* 93:787–796.

Fig. S1-1. ML tree based on harvested 18S rDNA data

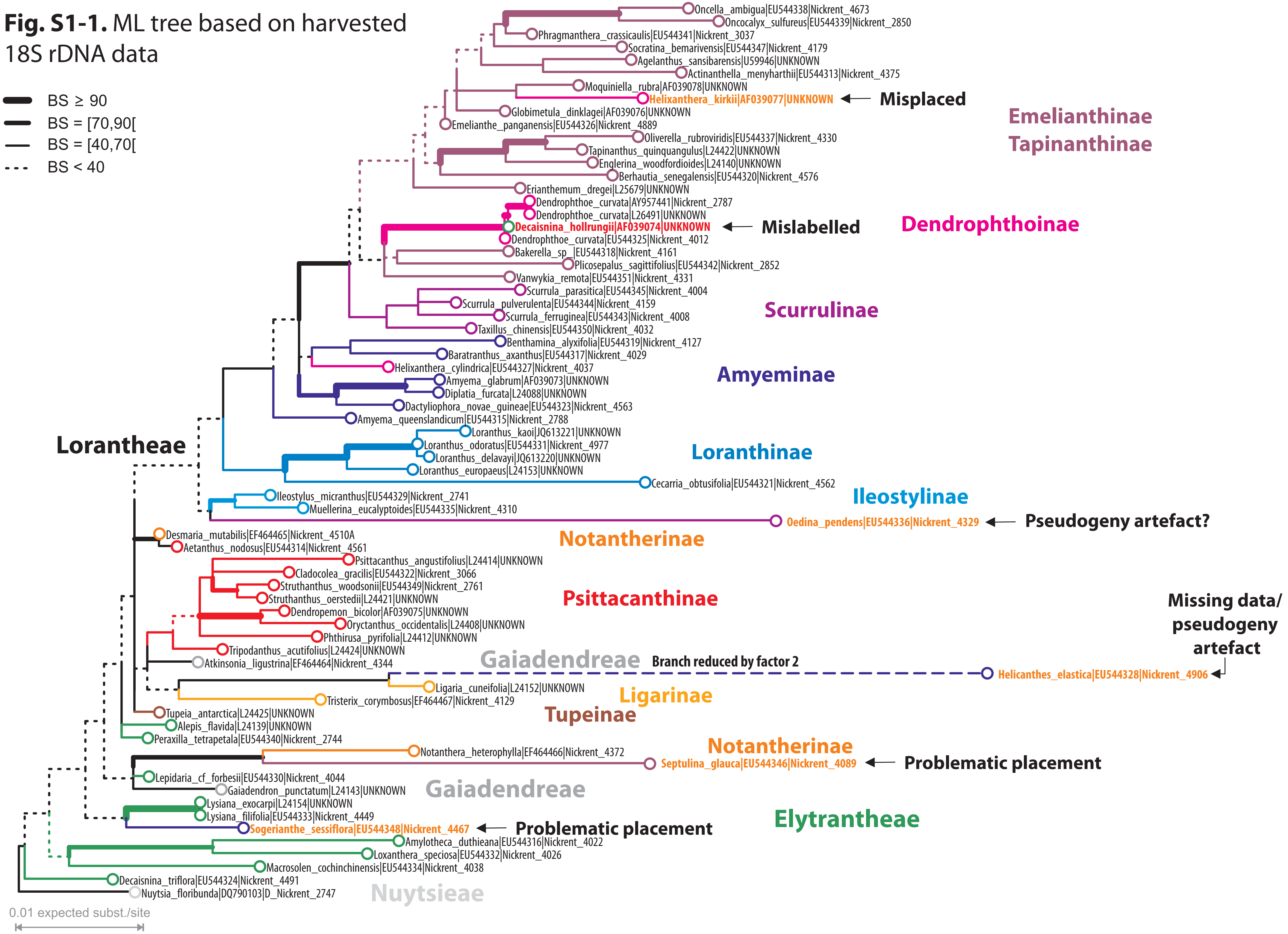

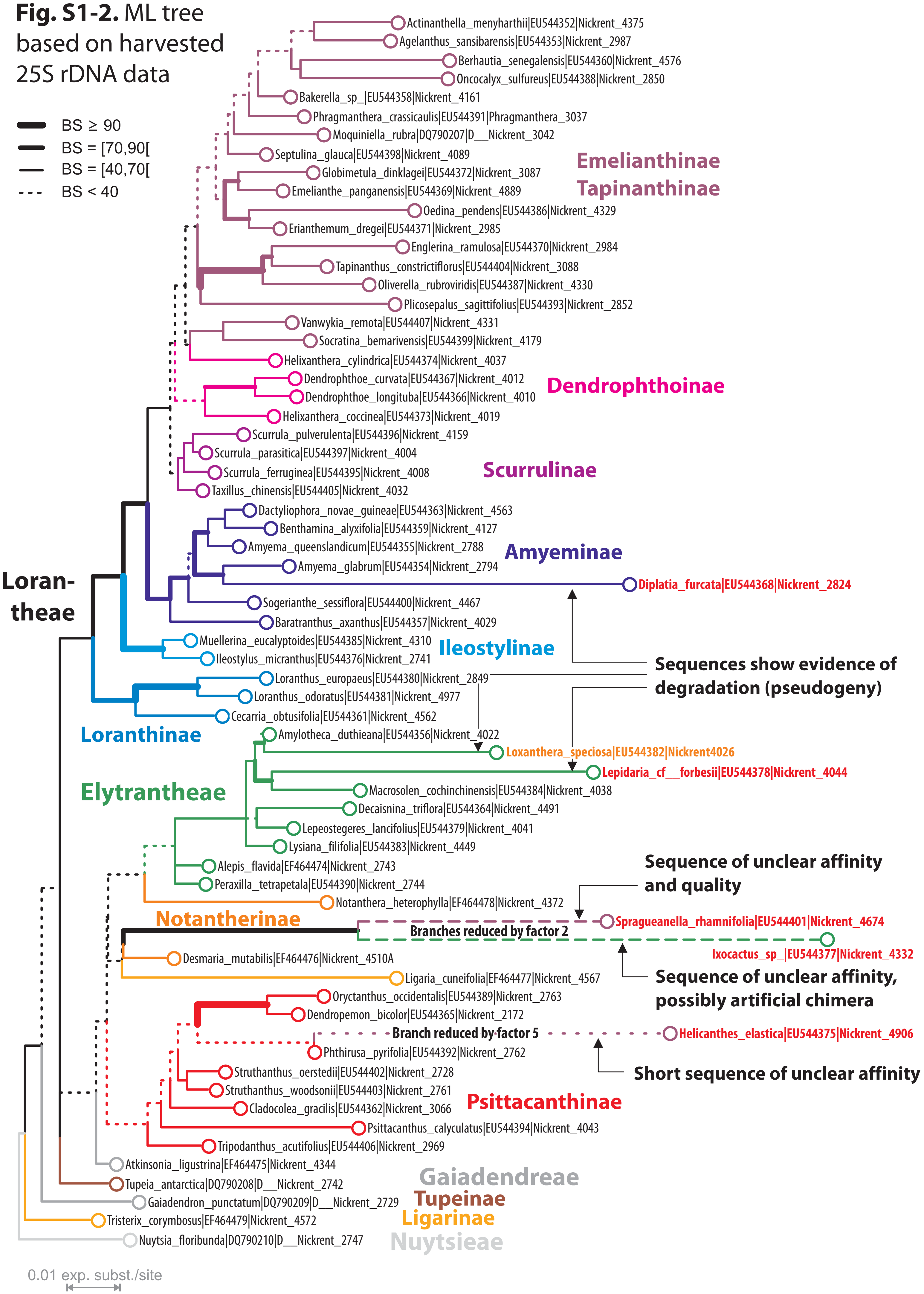

Fig. S1-2. ML tree based on harvested 25S rDNA data

Fig. S1-3. ML tree based on harvested *mat*K data

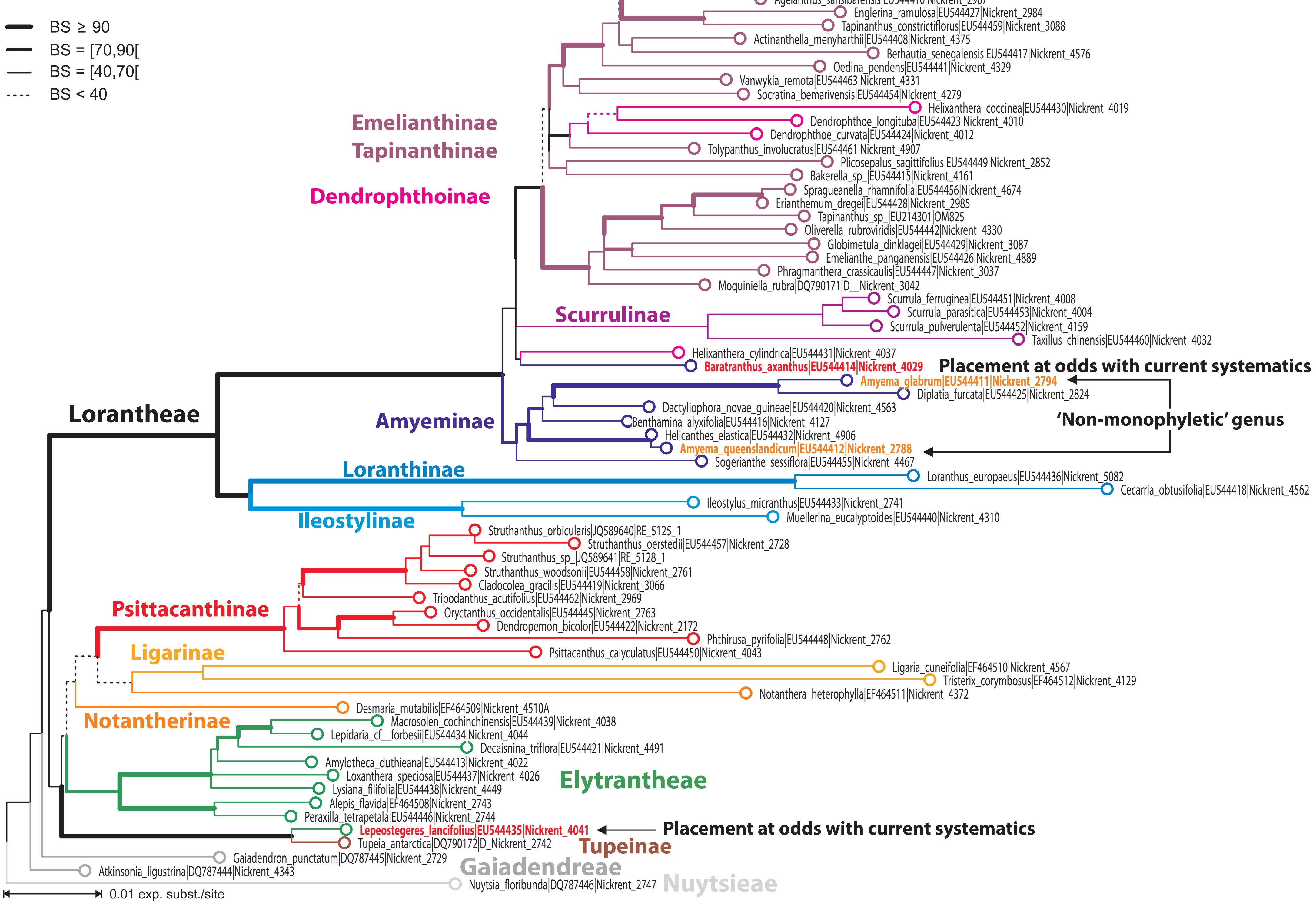

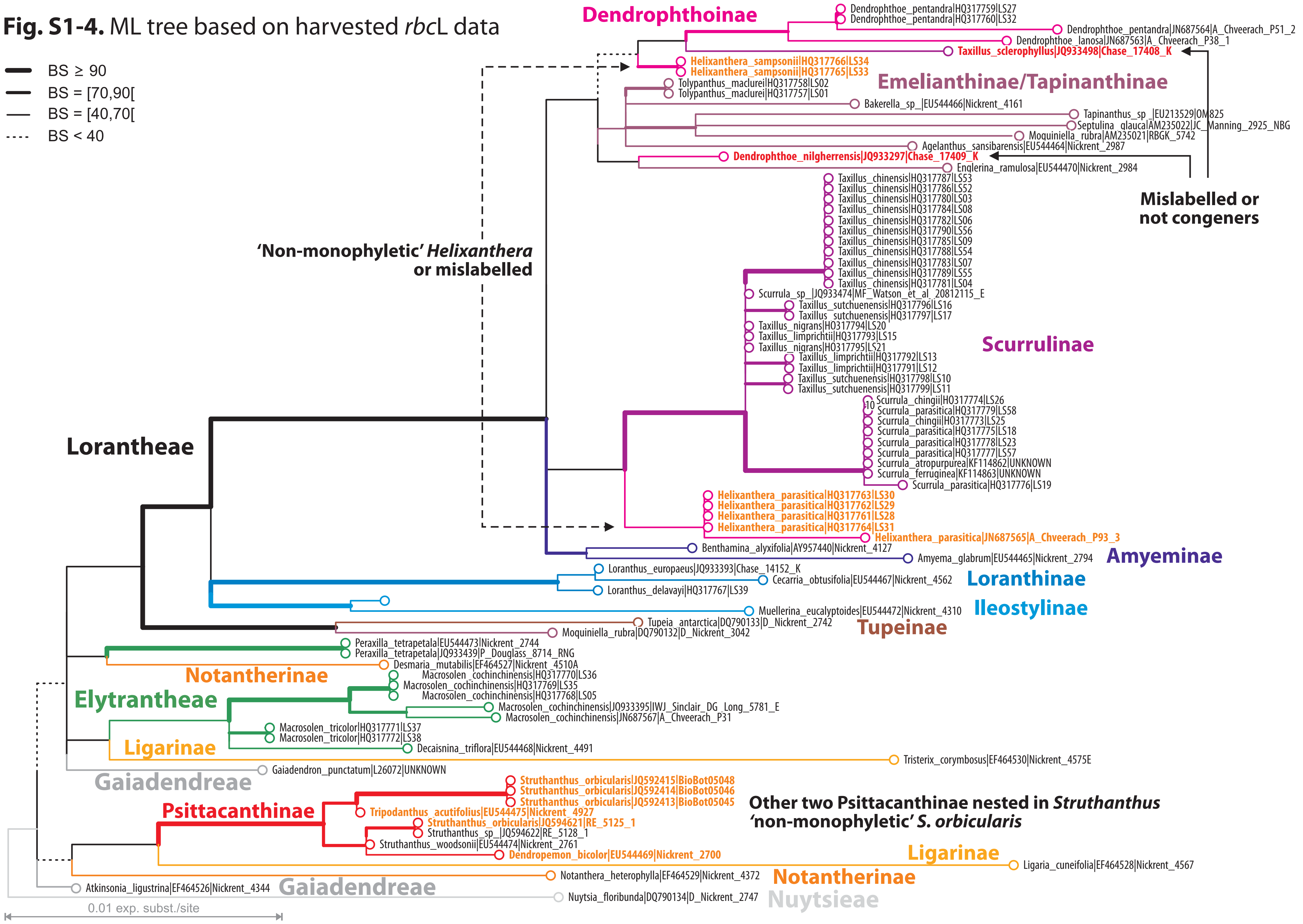

Fig. S1-4. ML tree based on harvested *rbc*L data

Fig. S1-5. ML tree based on harvested trnLLF data

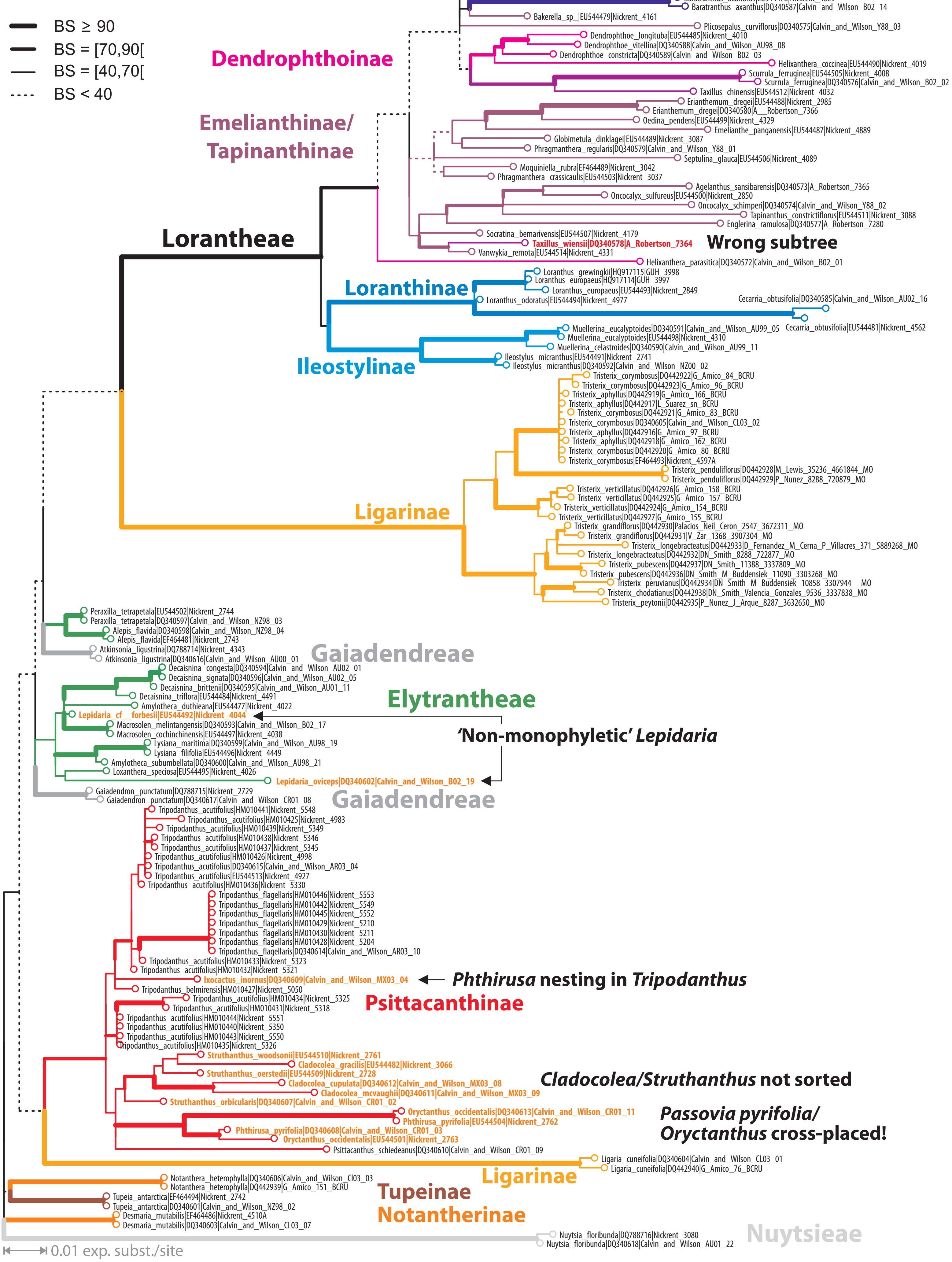

Supplement: Supplemental Information 1 — This supplement details the steps of the data harvesting, curating, and analyses and includes an annotated tabulation of used node height priors for each scenario as well as comprehensive single-gene maximum likelihood trees inferred from the unfiltered harvested data. [file peerj-05-3373-s001.pdf]
