# Supplementary material for: Eocene Loranthaceae pollen pushes back divergence ages for major splits in the family
Source: PeerJ. 2017 Jun 7;5:e3373. doi: 10.7717/peerj.3373 (PMC5466002; doi:10.7717/peerj.3373)
Supplement: Supplemental Information 3 — This file includes Plates S01 to S16 referenced in the main text showing high-resolution LM and SEM micrographs (overviews and details) of the newly described fossil pollen. [file peerj-05-3373-s003.pdf]

**Grímsson et al. File Sx: Supplementary Plate S1**

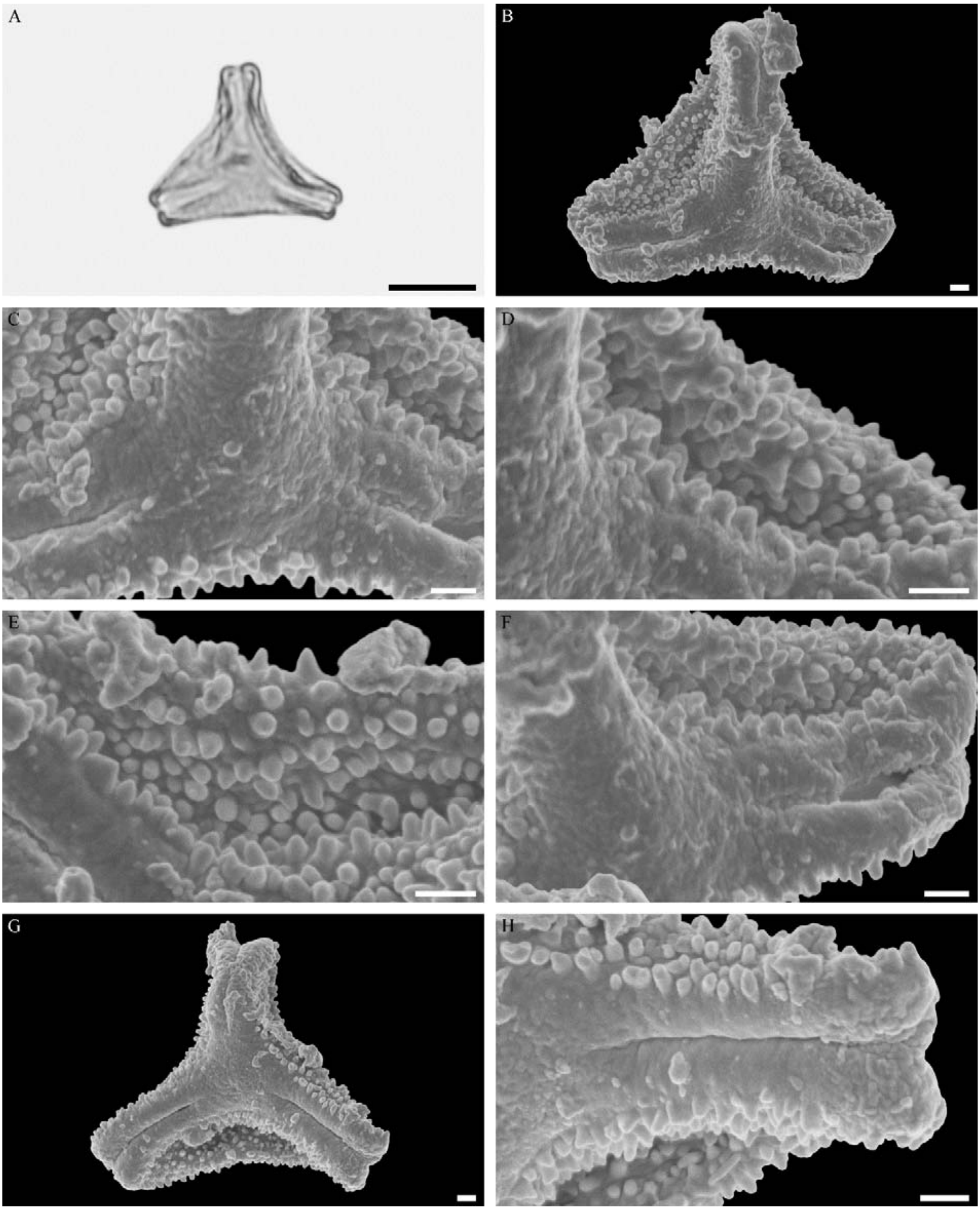


(See next page for legend)

## Grímsson et al. File Sx: Supplementary Plates S1 (legend) and S2

**Plate S01 (preceding page).** Fossil Loranthaceae pollen from the middle Eocene Claiborne Group, Tennessee, United States. (A) LM micrograph. (B–H) SEM micrographs. (A–H) Miller Clay Pit MT1. (A) Polar view, zono(3)colpate grain, nexine thickened along colpi. (B) Polar view, concave triangular grain. (C). Close-up showing polar area. (D) Close up showing echini in area of mesocolpium. (E) Close-up showing echini in area of mesocolpium. (F) Close-up showing equatorial apex and psilate to slightly rugulate margo around colpi. (G) Polar view, other side of grain figured in panel B. (H) Close up showing equatorial apex. Scale bars: (A) = 10 µm; (B–H) = 1 µm.

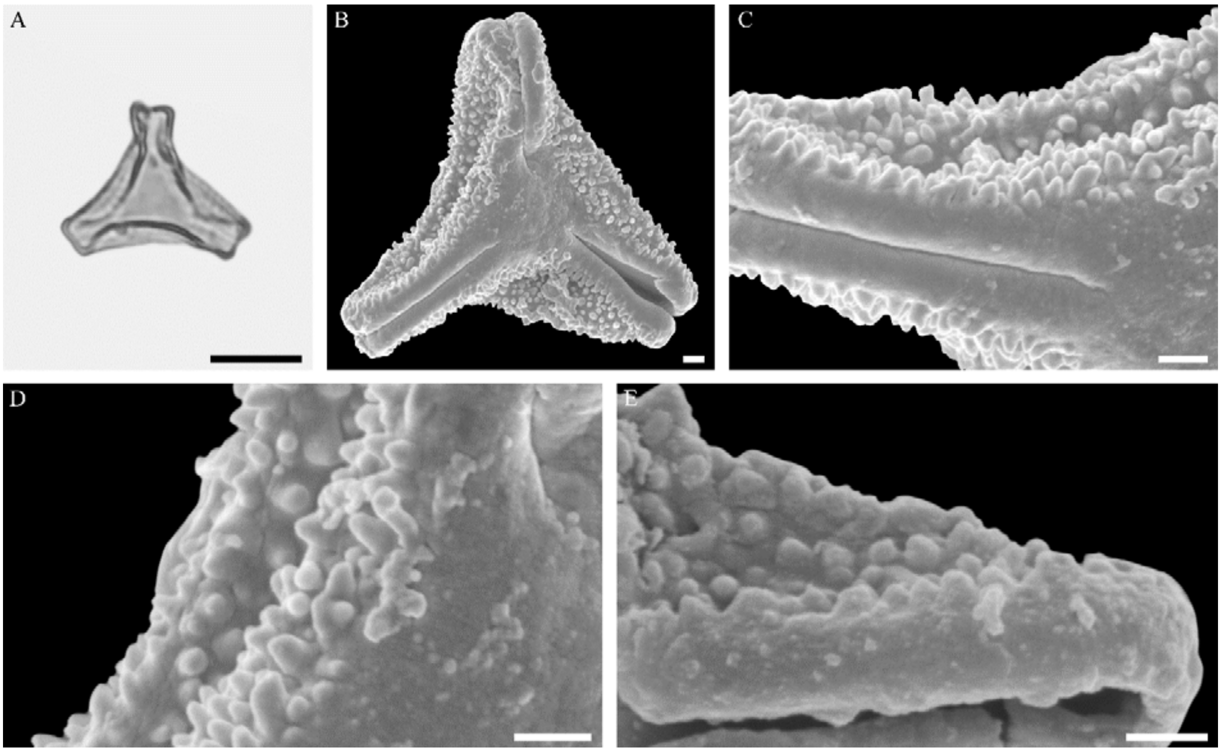


**Plate S02.** Fossil Loranthaceae pollen from the middle Eocene Claiborne Group, Tennessee, United States. (A) LM micrograph. (B–E) SEM micrographs. (A–E) Miller Clay Pit MT1. (A) Polar view, zono(3)colpate grain. (B) Polar view, triangular grain. (C Close-up of equatorial apex showing psilate margo along colpi and echinate sculpturing in area of mesocolpium. (D) Close-up showing central polar area and echini in area of mesocolpium. (E) Other side of pollen figured in panel B, close-up showing broad based echini in area of mesocolpium. Scale bars: (A) = 10 µm; (B–E) = 1 µm.

**Grímsson et al. File Sx: Supplementary Plate S3**

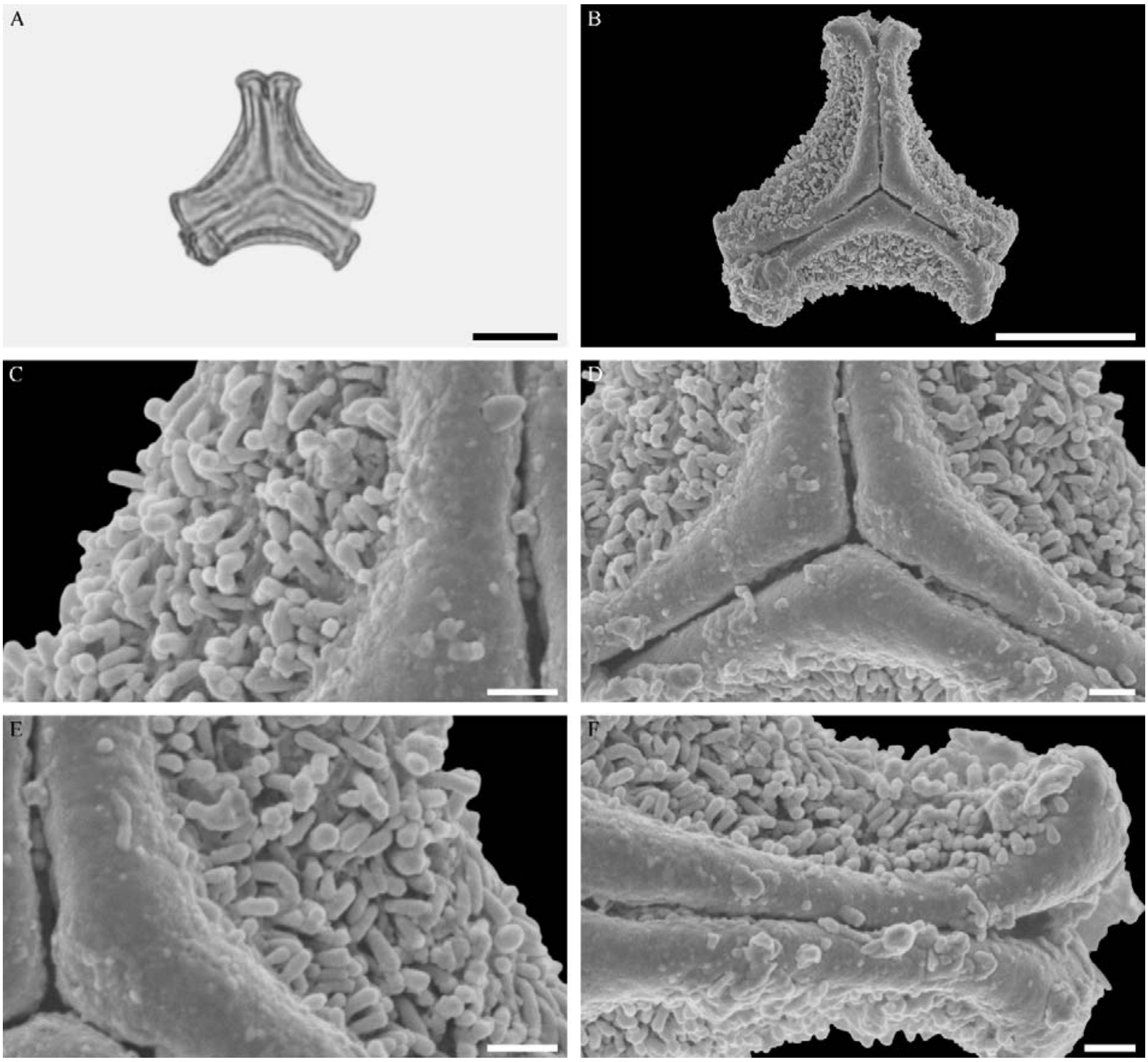


**Plate S03.** Fossil Loranthaceae pollen from the middle Eocene Claiborne Group, Tennessee, United States. (A) LM micrograph. (B–F) SEM micrographs. (A–F) Miller Clay Pit MT2. (A) Polar view, syn(3)colpate grain. (B) Polar view, concave-triangular grain with truncate to anchor-like apices. (C). Close-up showing (micro)baculae in area of mesocolpium. (D) Close-up of polar area, broad margo. (E) Close-up showing (micro)baculae in area of mesocolpium. (F) Close-up of equatorial apex, margo narrow, equatorial apex anchor-like. Scale bars: (A, B) = 10 µm; (C–F) = 1 µm.

**Grímsson et al. File Sx: Supplementary Plate S4**

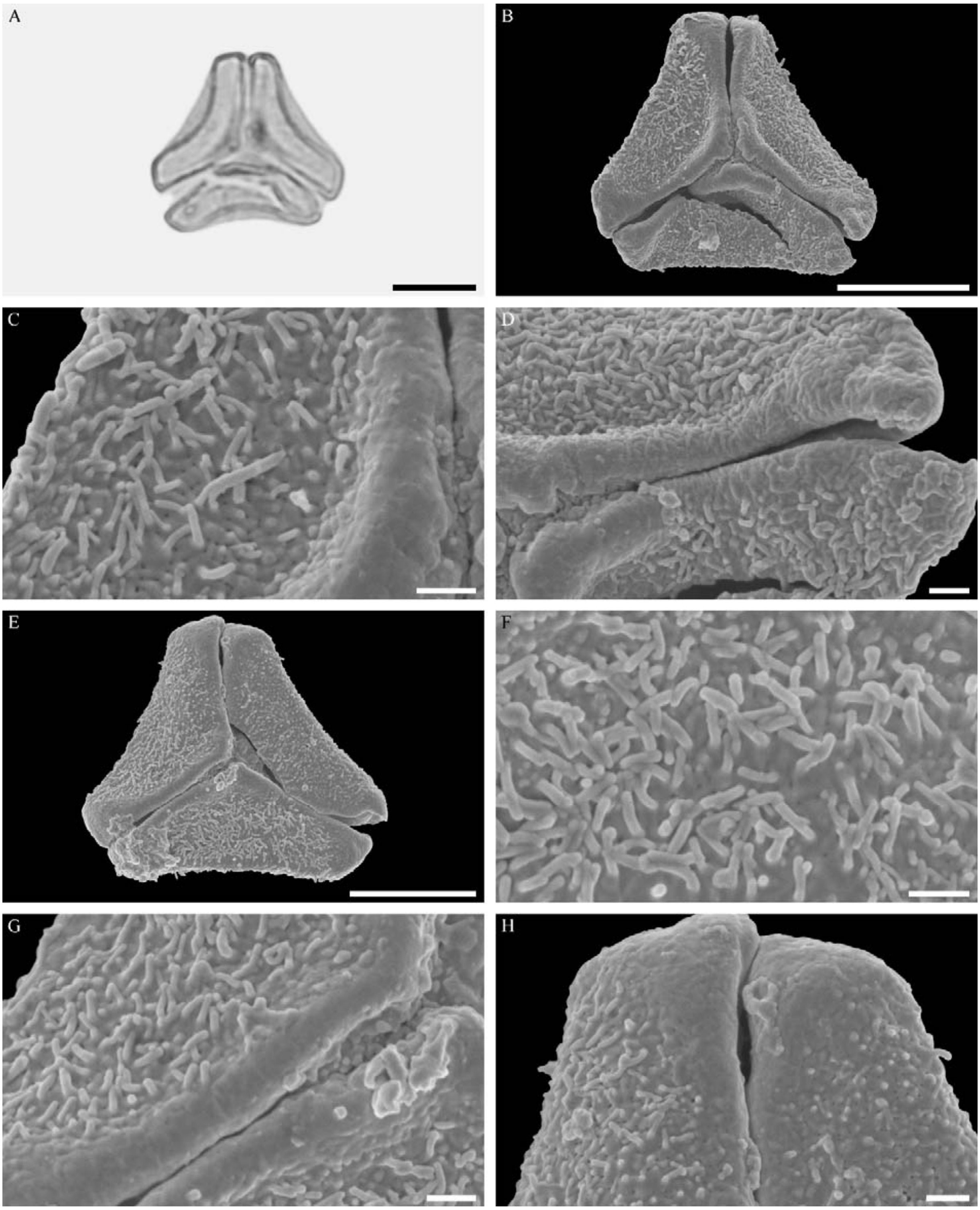


(See next page for legend)

## Grímsson et al. File Sx: Supplementary Plates S4, S5 (legends), and S6

**Plate S04 (preceeding page).** Fossil Loranthaceae pollen from the middle Eocene Claiborne Group, Tennessee, United States. (A) LM micrograph. (B–H) SEM micrographs. (A–H) Miller Clay Pit MT3. (A) Polar view, syn(3)colpate grain. (B) Polar view, slightly concave-triangular grain with truncate apices. (C). Close-up showing (micro)baculate and perforate sculpturing in area of mesocolpium. (D) Close-up of equatorial apex, microrugulate margo around colpi. (E) Polar view, other side of grain figured in panel B. (F) Close-up showing (micro)baculae in area of mesocolpium. (G) Close-up showing colpus membrane, narrow psilate margo around colpi, and (micro)baculae in area of mesocolpium. (H) Close-up of truncated equatorial apex, margo broad. Scale bars: (A, B, E) = 10 µm; (C, D, F–H) = 1 µm.

**Plate S05 (next page).** Fossil Loranthaceae pollen from the middle Eocene Hareøen Formation, Qeqertarsuatsiaq Island, western Greenland. (A) LM micrograph. (B-H) SEM micrographs. (A–H) Aamaruutissaa MT. (A) Polar view, syn(3)colpate grain, nexine thickened along colpi. (B) Polar view, triangular grain with truncated equatorial apices. (C) Close-up showing (micro)baculae in area of mesocolpium. (D) Close-up showing (micro)baculae in area of mesocolpium. (E) Polar view, other side of grain figured in panel B. (F) Close-up showing microrugulate to microverrucate margo in polar area. (G) Close-up, equatorial apex. (H) Close-up showing (micro)baculae in area of mesocolpium. Scale bars: (A, B, E) = 10 µm; (C, D, F–H) = 1 µm.

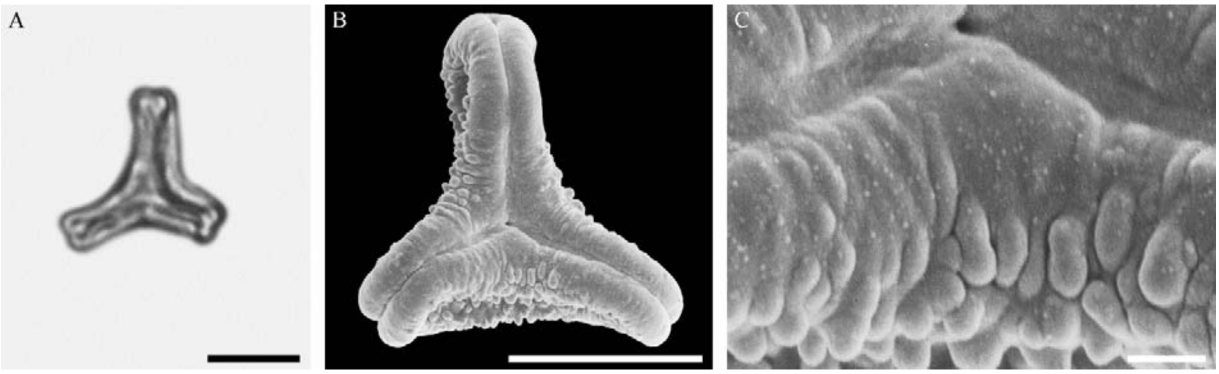


**Plate S06.** Fossil Loranthaceae pollen from the middle Eocene Borkener coal measures, Kassel, Germany. (A) LM micrographs. (B, C) SEM micrographs. (A–C) Stolzenbach MT. (A) Polar view, syn(3)colpate grain. (B) Polar view, concave triangular grain. (C) Close-up showing psilate to slightly rugulate margo and broadly based echini in area of mesocolpium. Scale bars: (A, B) = 10 µm; (C) = 1 µm.

**Grímsson et al. File Sx: Supplementary Plate S5**

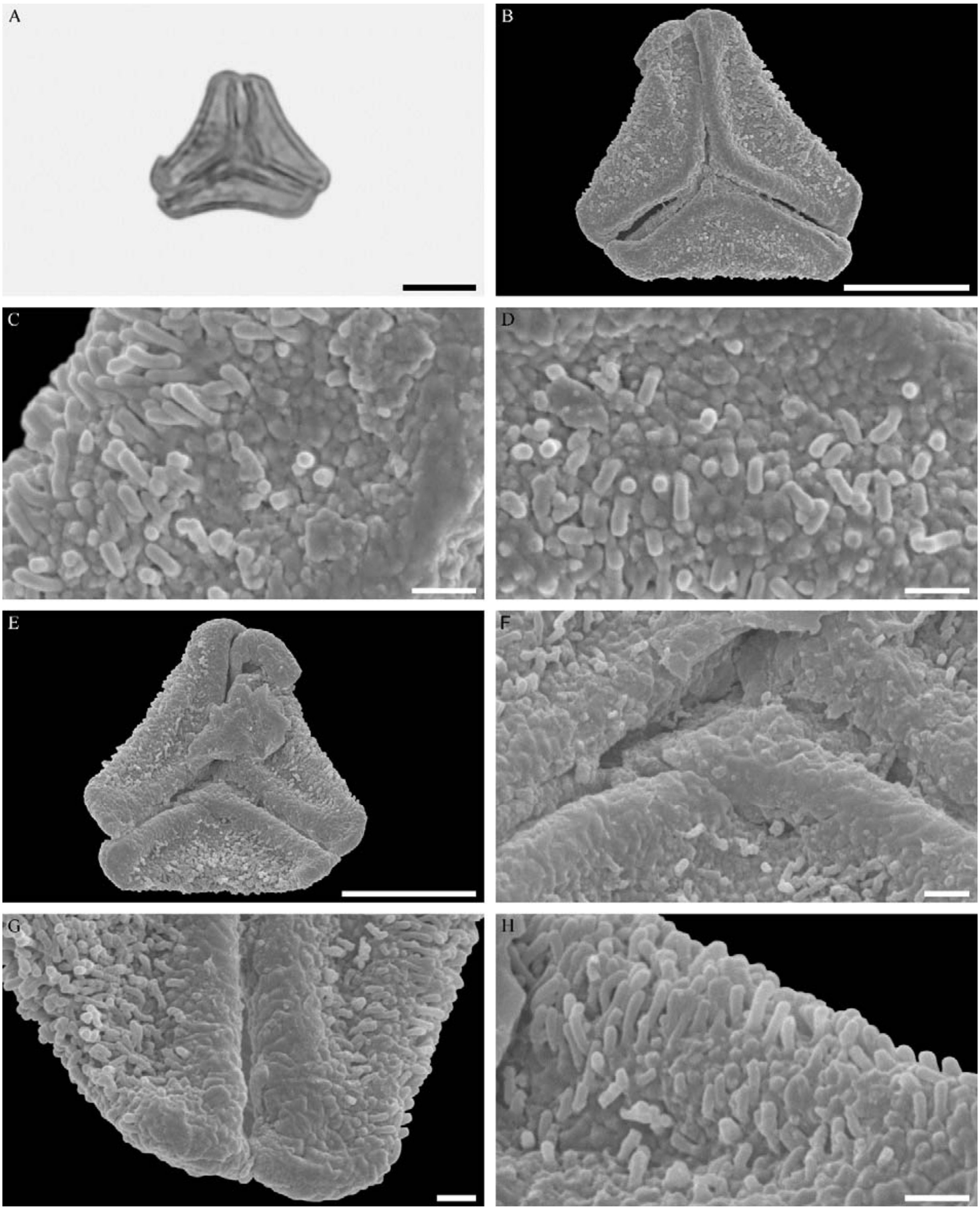


(See preceding page for legend)

**Grímsson et al. File Sx: Supplementary Plate S7**

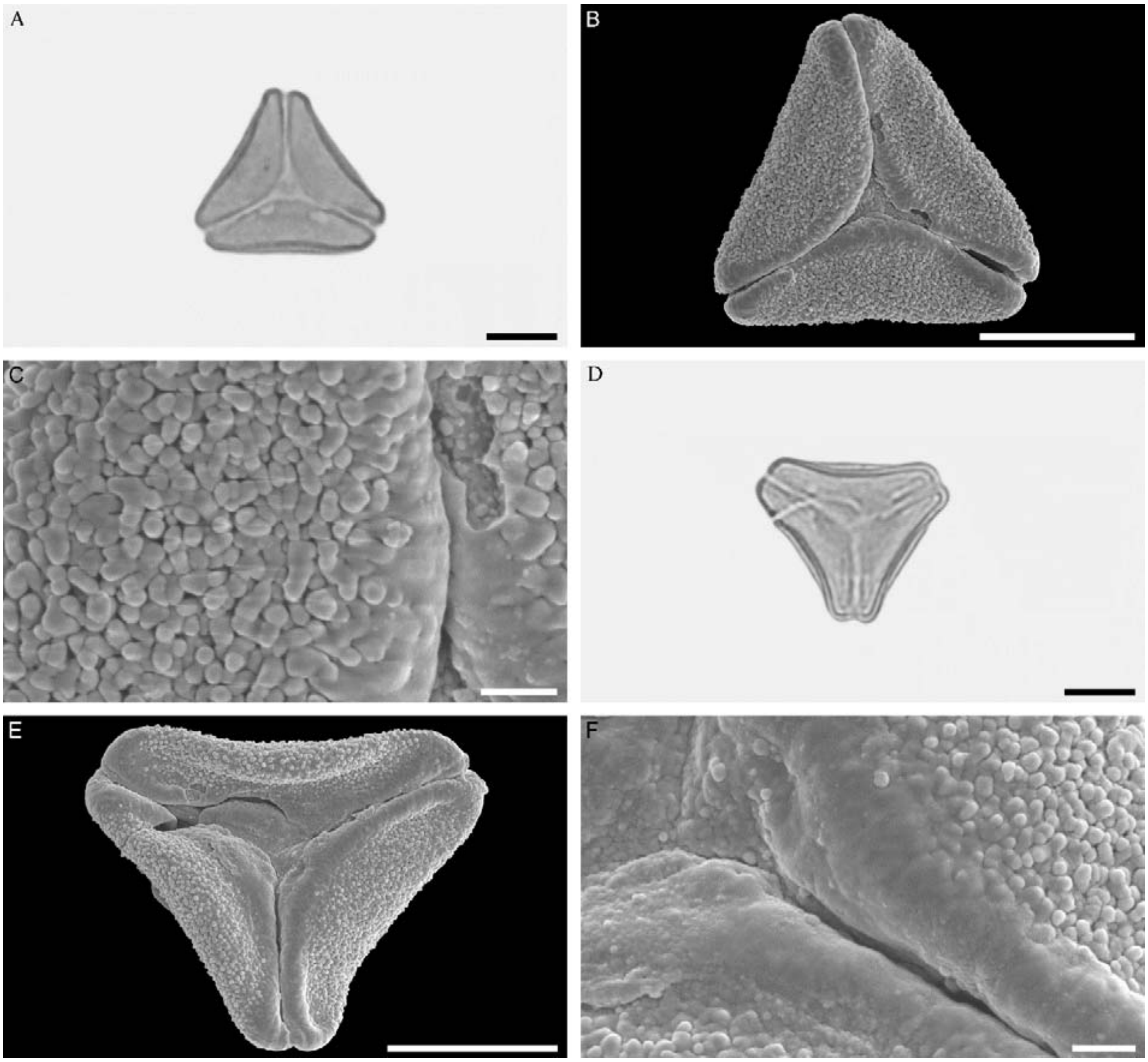


**Plate S07.** Fossil Loranthaceae pollen from the middle Eocene Profen Formation, Germany. (A, D) LM micrographs. (B, C, E–J) SEM micrographs. (A–C) Profen MT1. (A) Polar (upper) and equatorial (lower) views, trilobate grain. (B) Equatorial view, grain elliptic. (C) Close-up showing nanoechinate to nanobaculate, granulate sculpturing in area of mesocolpium. (D–H) Profen MT1. (D) Polar (upper), oblique (middle) and equatorial (lower) views. (E) Polar view, syn(3)colpate grain. (F) Close-up showing nanoechinate to nanobaculate sculpturing in area of mesocolpium. (G) Close-up showing central polar area. (H) Close up of apex and margo. (I, J) Profen MT1. (I) Pollen in polar view, trilobate grain. (J). Close-up showing central polar area and nanoechinate to nanobaculate sculpturing along margo and in area of mesocolpium. Scale bars: (A, D) = 10 µm; (B, C, E-J) = 1 µm.

# Grímsson et al. File Sx: Supplementary Plate S8

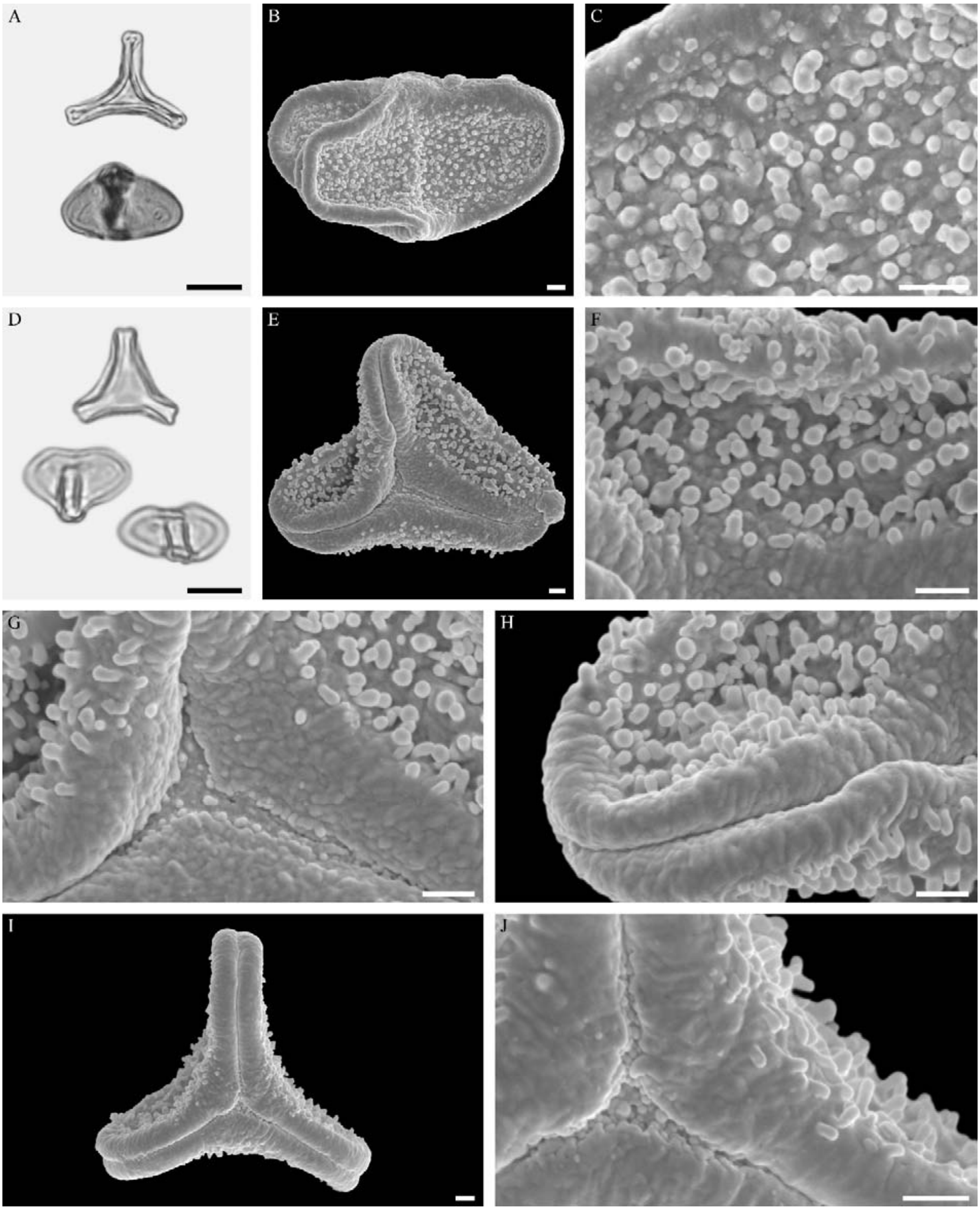


(See next page for legend)

## Grímsson et al. File Sx: Supplementary Plates S8 (legend), S9, and S10 (legend)

**Plate S08 (preceeding page).** Fossil Loranthaceae pollen from the middle Eocene Profen Formation, Germany. (A, D) LM micrographs. (B, C, E, F) SEM micrographs. (A–C) Profen MT2. (A) Polar view, intercolpial nexine thickenings at pole. (B) Polar view, triangular syn(3)colpate grain. (C) Close-up showing nanoechinate/-baculate, perforate sculpturing in area of mesocolpium. (D–F) Profen MT2. (D) Polar view, intercolpial nexine thickening at pole. (E) Polar view, triangular syn(3)colpate grain with truncated equatorial apices. (F) Close-up showing central polar area, psilate margo and nanoechini/-baculae in area of mesocolpium. Scale bars: (A, B, D, E) = 10 µm; (C, F) = 1 µm.

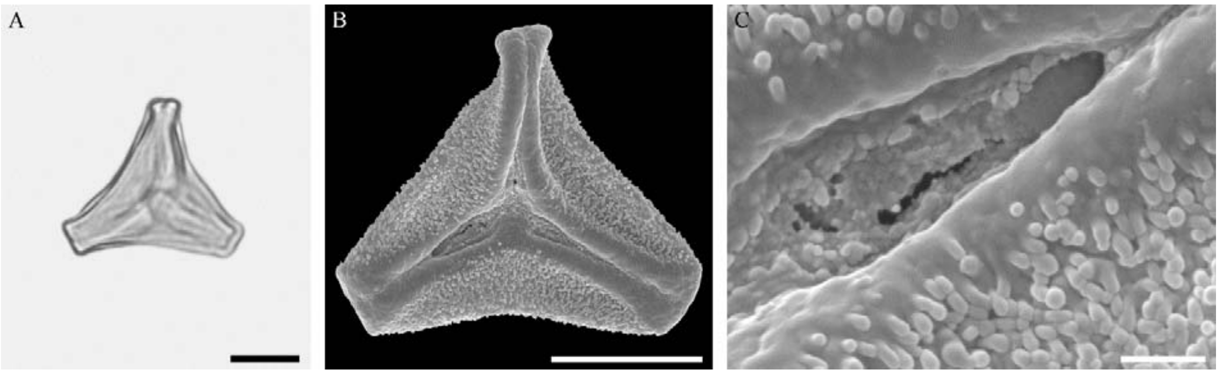


**Plate S09.** Fossil Loranthaceae pollen from the middle Eocene Profen Formation, Germany. (A) LM micrograph. (B, C) SEM micrographs. (A–C) Profen MT3. (A) Polar view, convex-triangular grain. (B) Polar view, syn(3)colpate grain. (C) Close-up showing colpus membrane, margo and sculpting elements in area of mesocolpium. Scale bars: (A, B) = 10 µm; (C) = 1 µm.

**Plate S10 (following page).** Fossil Loranthaceae pollen from the middle Eocene Profen Formation, Germany. (A) LM micrograph. (B–H) SEM micrographs. (A–H) Profen MT4. (A) Polar view, nexine hexagonally thickened in polar area. (B) Polar view, concave triangular demisyn(3)colpate grain with anchor-like equatorial apices. (C) Close-up of colpus membrane in central polar area. (D) Close-up of equatorial apex, colpi short. (E) Close-up of sculpturing elements in area of mesocolpium. (F) Polar view, other side of grain figured in panel B. (G) Close-up of colpus membrane in polar area and sculpturing along margo. (H) Close-up showing nanobaculate/-echinate sculpturing in area of mesocolpium. Scale bars: (A, B) = 10 µm; (C–H) = 1 µm.

**Grímsson et al. File Sx: Supplementary Plate S10**

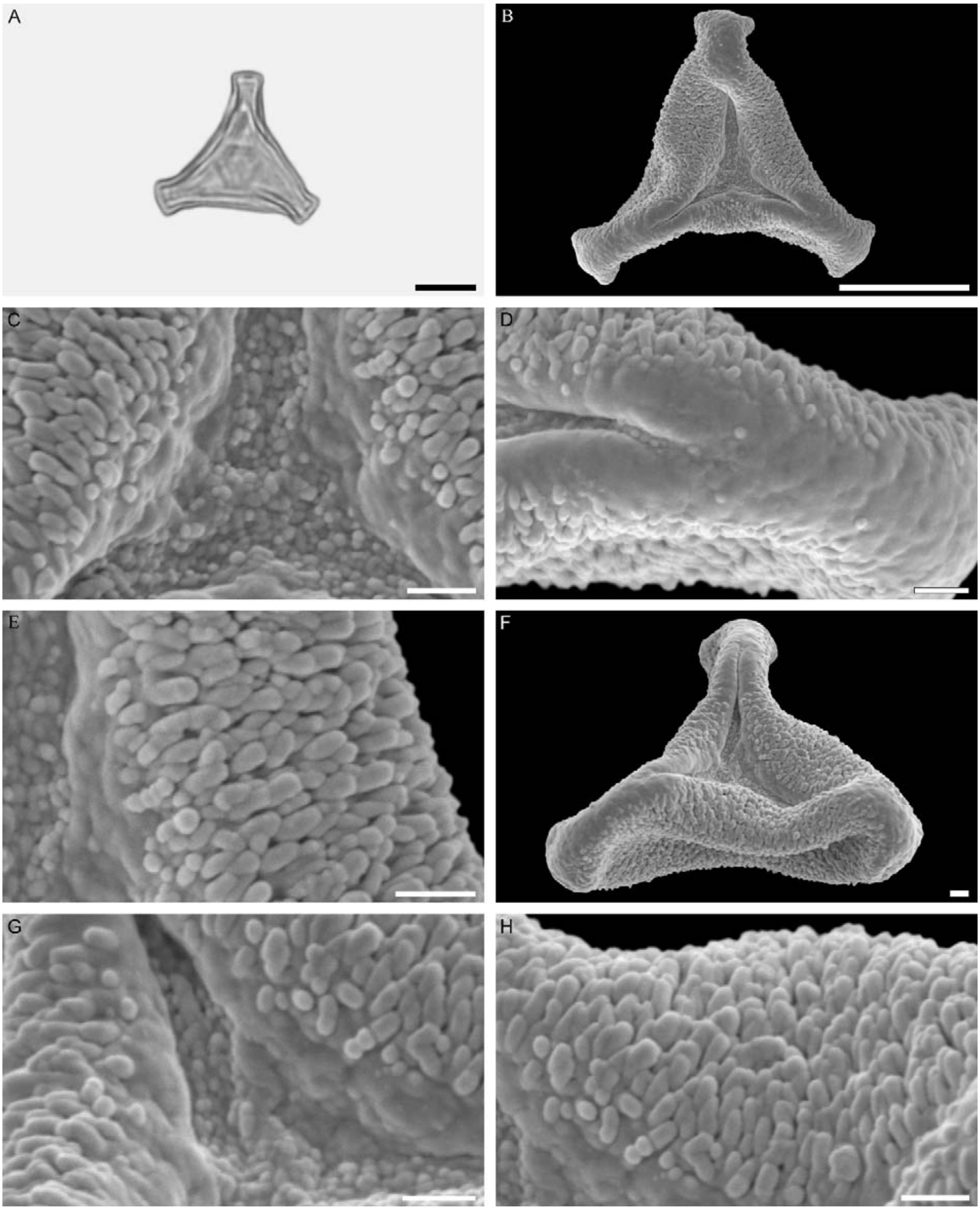


(See preceding page for legend)

**Grímsson et al. File Sx: Supplementary Plate S11**

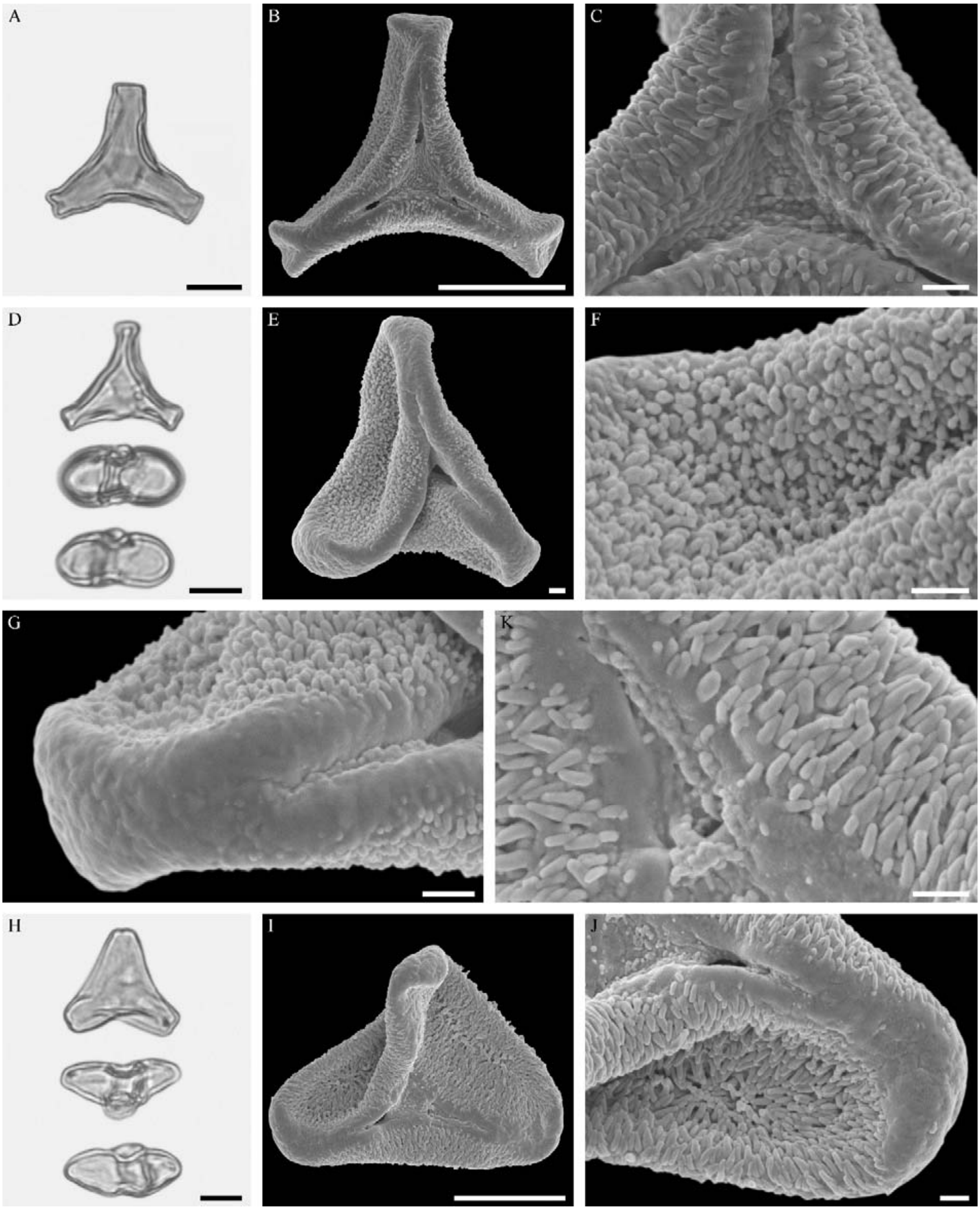


(See next page for legend)

**Grímsson et al. File Sx: Legends for Supplementary Plates S11–S13**

**Plate S11 (preceding page).** Fossil Loranthaceae pollen from the middle Eocene Profen Formation, Germany. (A, D, I) LM micrographs. (B, C, E, F, G, H, J, K) SEM micrographs. (A–C) Profen MT4. (A) Polar view, nexine hexagonally thickened in polar area. (B) Polar view, concave triangular demisyn(3)colpate grain with anchor-like equatorial apices. (C) Close-up showing polar area. (D–G) Profen MT4. (D) Polar (upper) and equatorial (middle, lower) views, nexine hexagonally thickened in polar area. (E) Polar view, demisyn(3)colpate grain. (F) Close up showing sculpturing in area of mesocolpium. (G) Close-up of apex. (H–K) Profen MT4. (H) Polar (upper) and equatorial (middle, lower) views, nexine hexagonally thickened in polar area. (I) Polar view, demisyn(3)colpate grain. (J) Close-up of apex, colpi short. (K) Close-up of polar area showing colpus membrane, margo and sculpturing in area of mesocolpium. Scale bars: (A, B, D, H, I) = 10 µm; (C, E-G, J, K) = 1 µm.

**Plate S12 (following page).** Fossil Loranthaceae pollen from the middle Eocene Profen Formation, Germany. (A, E) LM micrographs. (B–D, F–H) SEM micrographs. (A–D) Profen MT5. (A) Polar (left) and equatorial (right) views. (B) Polar view, triangular demisyn(3)colpate grain. (C) Close-up showing polar area. (D) Close-up showing nano- to microbaculate/-echinate sculpturing in area of mesocolpium. (E-H) Profen MT5. (E) Polar (left) and equatorial (right) views, grain distorted/compressed in equatorial view. (F) Polar view, triangular demisyn(3)colpate grain. (G) Close-up showing polar area, colpus membrane, margo and sculpturing in area of mesocolpium. (H) Close-up of apex. Scale bars: (A, B, E, F) = 10 µm; (C, D, G, H) = 1 µm.

**Plate S13 (p. 14).** Fossil Loranthaceae pollen from the middle Eocene Changchang Formation, South China. (A, H, J) LM micrograph. (B–G, I, K) SEM micrographs. (A–G) Changchang MT. (A) Polar view, two grains, nexine thickened along colpi. (B) Polar view, syn(3)colpate grains with truncated equatorial apices. (C) Other side of lower grain figured in panel B. (D) Close-up showing nanoverrucate to granulate sculpturing in area of mesocolpium. (E) Close-up of polar area showing granulate colpus membrane and psilate margo. (F) Close-up showing nanoverrucate to granulate sculpturing in area of mesocolpium. (G) Close-up showing triangular structures in equatorial apices similar to opercula. (H, I) Changchang MT. (H) Polar view, nexine thickened along colpi. (I) Polar view, grain with truncated equatorial apices. (J, K) Changchang MT. (J) Polar view, concave triangular grain, nexine thickened along colpi. (K) Polar view, concave triangular grain, truncated equatorial apices. Scale bars: (A–C, H–K) = 10 µm; (D–G) = 1 µm.

**Grímsson et al. File Sx: Supplementary Plate S12**

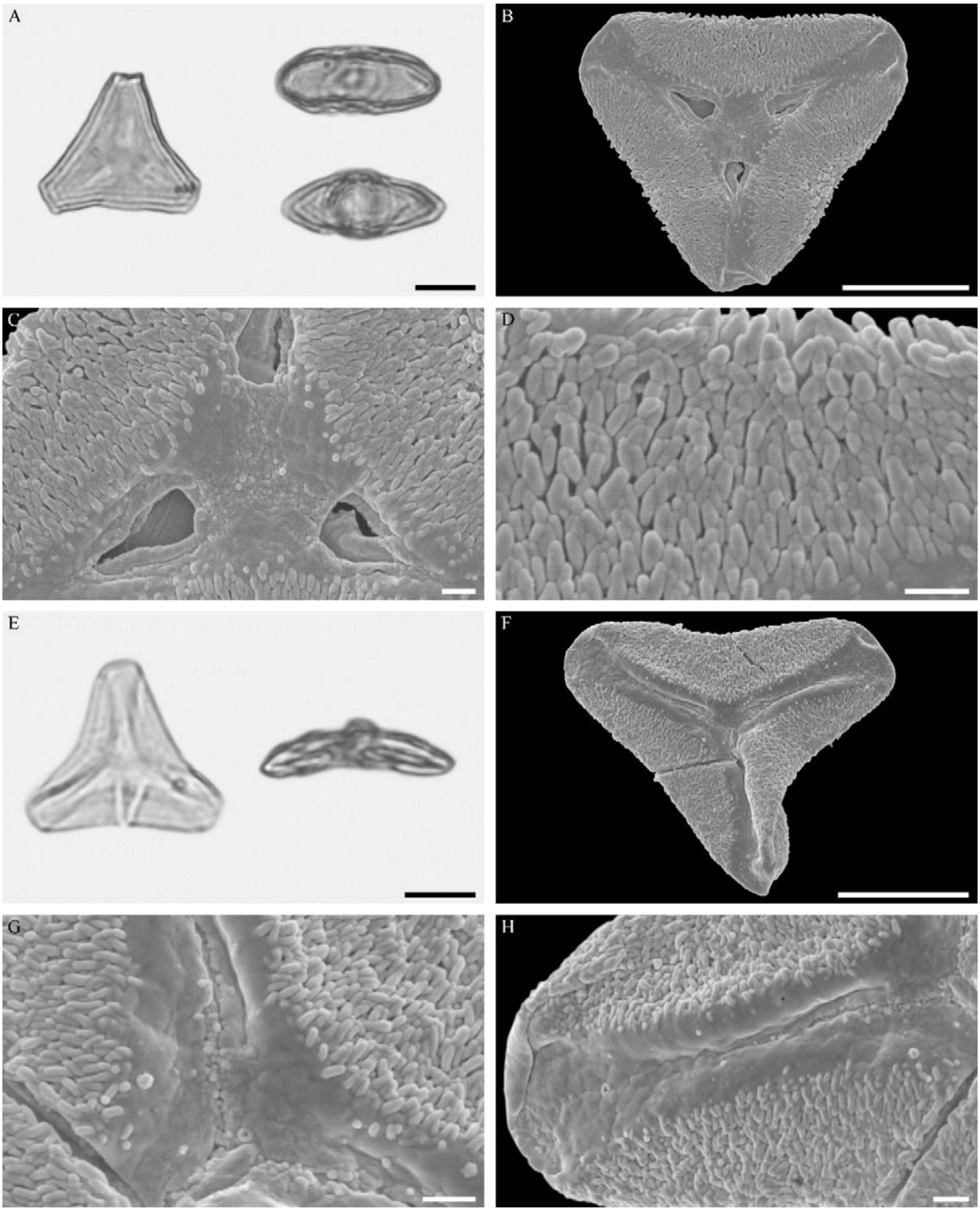


(See preceding page for legend)

**Grímsson et al. File Sx: Supplementary Plate S13**

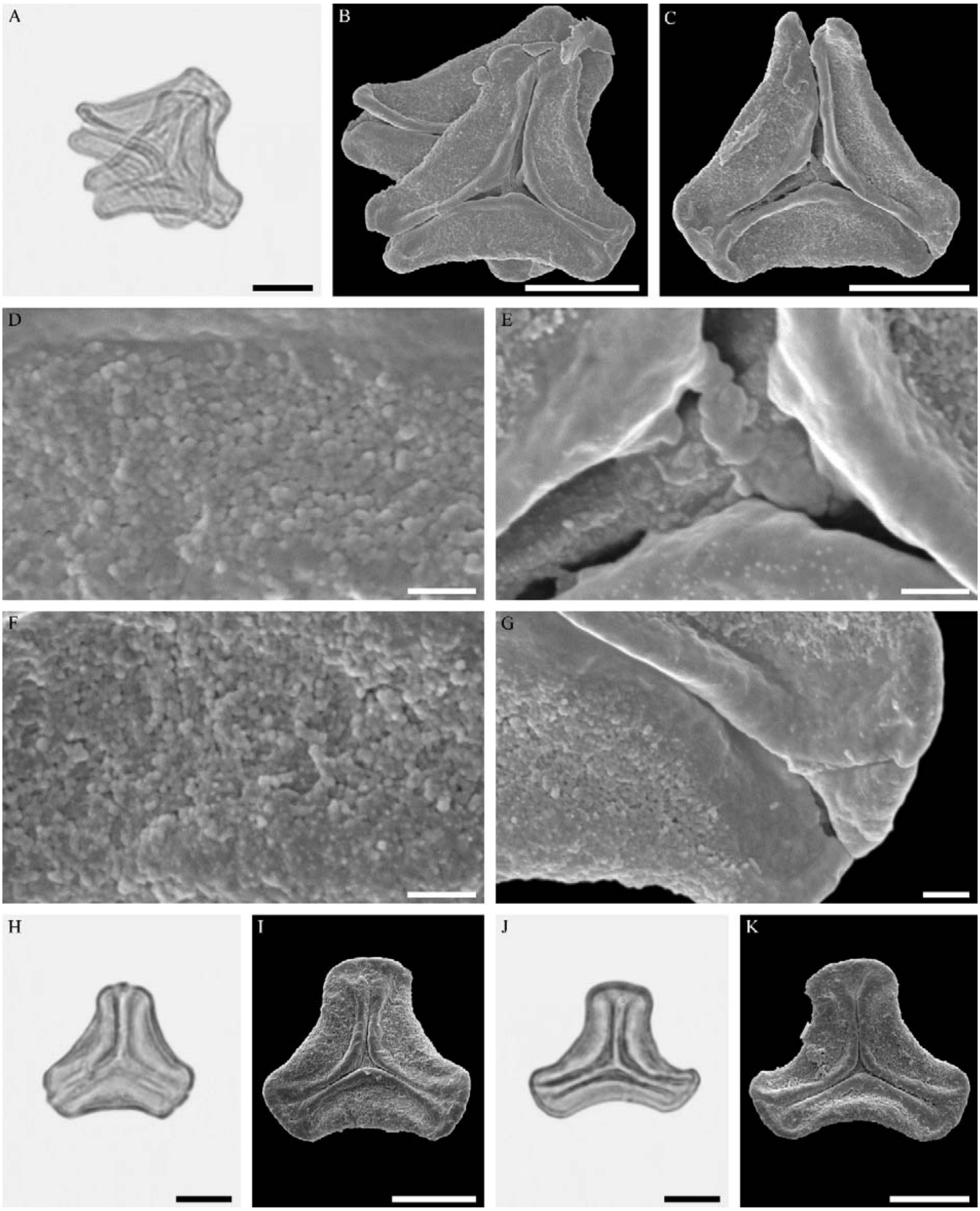


(See p. 12 for legend)

## Grímsson et al. File Sx: Supplementary Plate S14

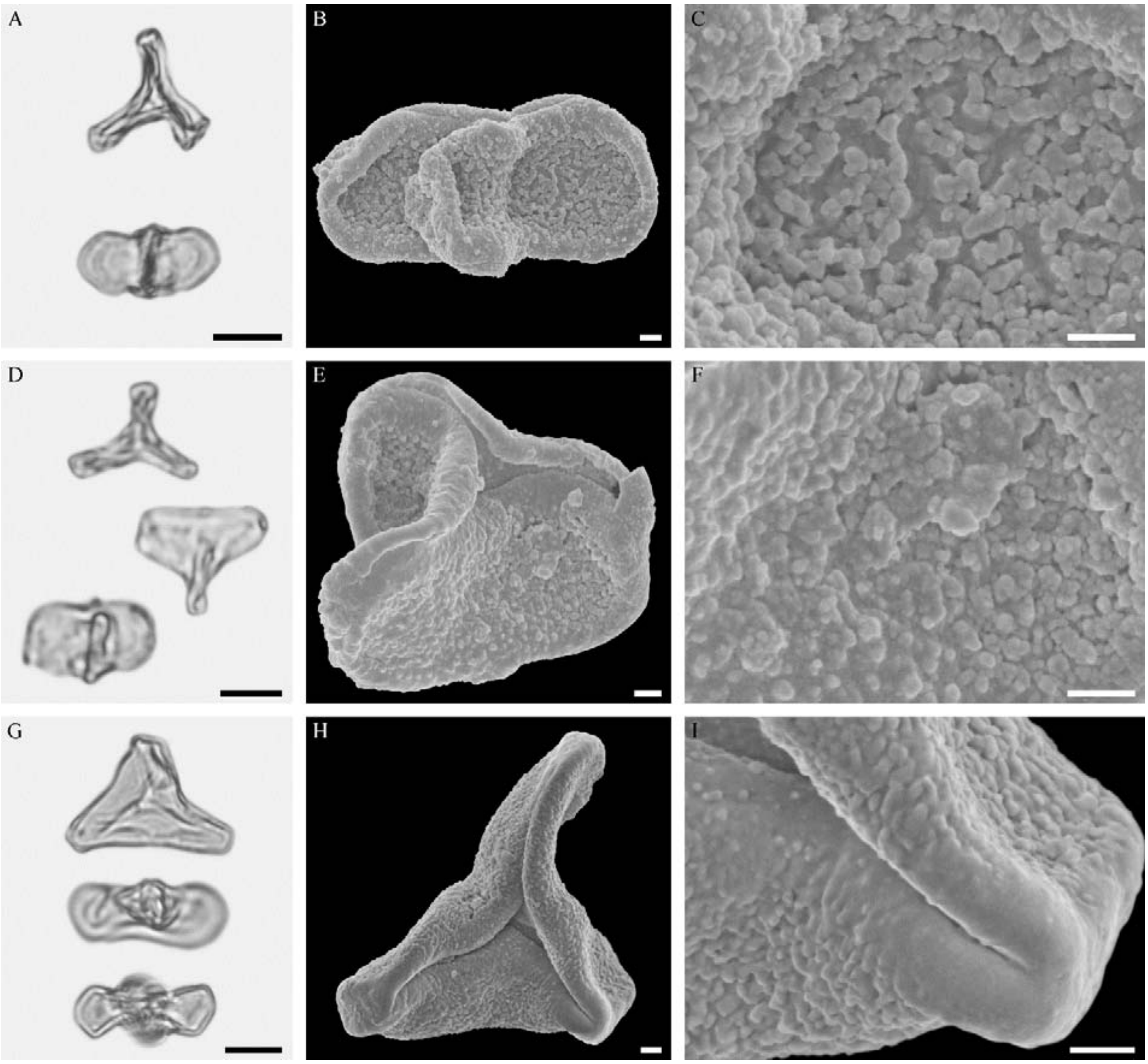


**Plate S14.** Fossil Loranthaceae pollen from the middle Oligocene Melker Series, Lower Austria. (A, D, G) LM micrographs. (B, C, E, F, H, I) SEM micrographs. (A–C) Theiss MT. (A) Polar (upper) and equatorial (lower) views, trilobate grain. (B) Equatorial view, slightly emarginate demisyn(3)colpate grain. (C) Close-up showing sculpturing in area of mesocolpium. (D–F) Theiss MT. (D) Polar (upper), oblique (middle) and equatorial (lower) view, trilobate grain. (E). Oblique polar view, demisyn(3)colpate grain, colpi long and extending towards equatorial margin of apex. (F) Close-up showing sculpturing in area of mesocolpium. (G) Polar (upper) and equatorial (middle/lower) views, triangular grain. (H) Poalr view, demisyn(3)colpate grain. (I) Close-up of apex. Scale bars: (A, D, G) = 10 µm; (B, C, E, F, H, I) = 1 µm.

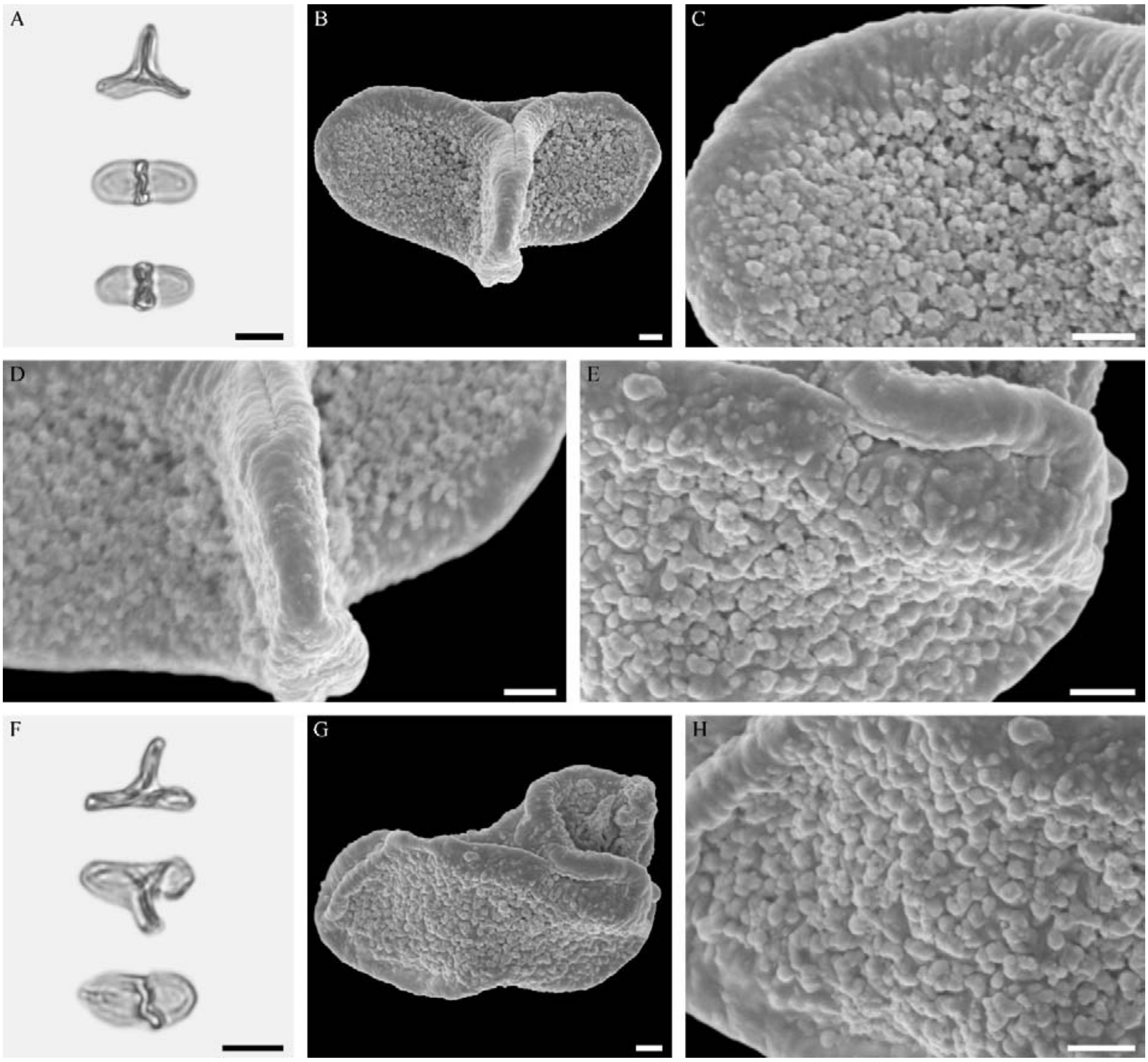


**Plate S15.** Fossil Loranthaceae pollen from the middle Oligocene Melker Series, Lower Austria. (A, F) LM micrographs. (B–E, G–H) SEM micrographs. (A–E) Theiss MT. (A) Polar (upper) and equatorial (middle/lower) views, trilobate grain. (B) Equatorial view, slightly emarginate demisyn(3)colpate grain. (C) Close-up showing sculpturing in area along margo and mesocolpium. (D) Close-up of apex showing colpi and sculpturing in area of mesocolpium. (F–H) Polar (upper), oblique (middle) and equatorial (lower) views, trilobate grain. (G) Oblique equatorial view, syn(3)colpate grain. (H) Close-up showing sculpturing in area of mesocolpium. Scale bars: (A, F) = 10 µm; (B–E, G, H) = 1 µm.

**Grímsson et al. File Sx: Supplementary Plate S16**

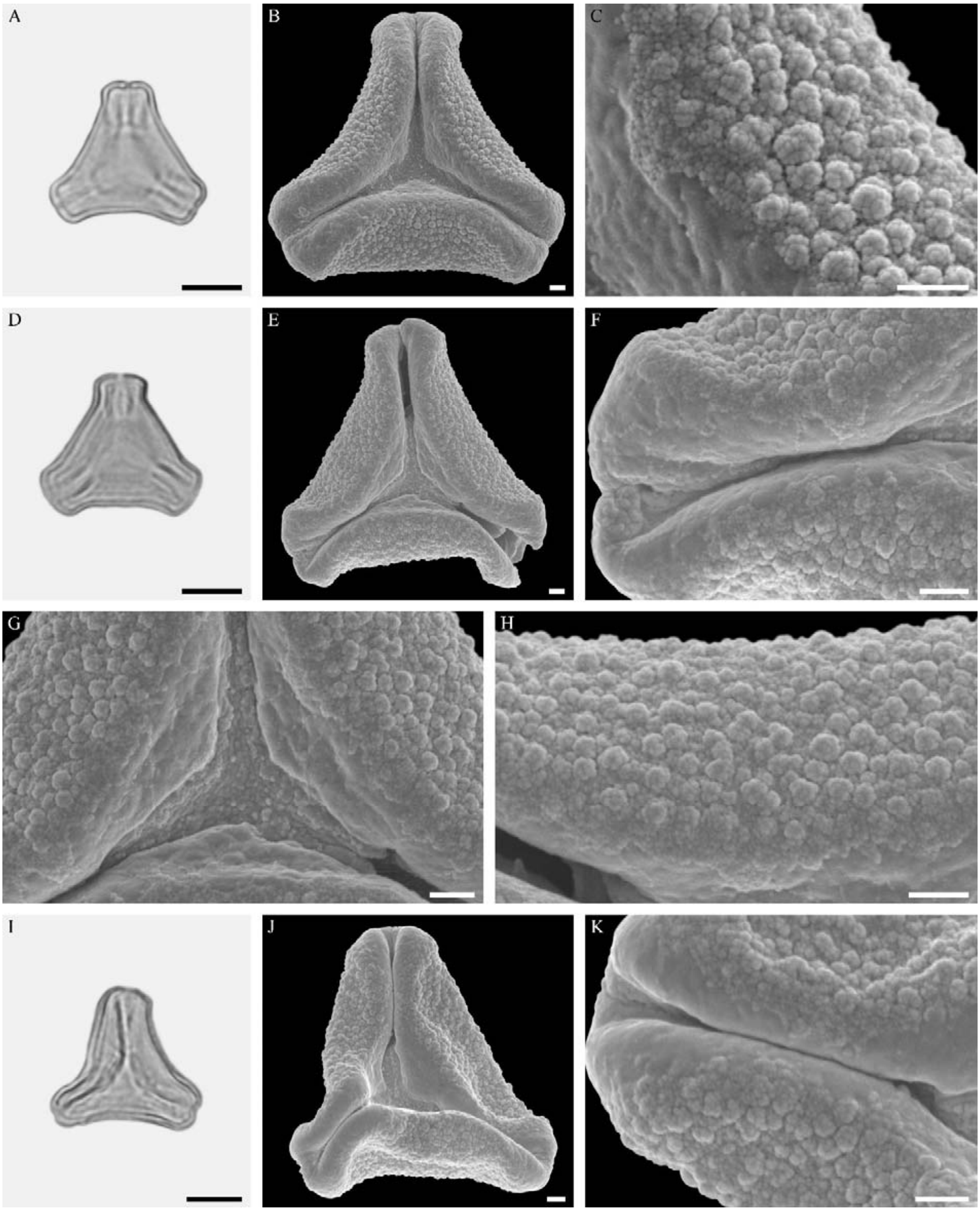


(See next page for legend)

## Grímsson et al. File Sx: Legend for Supplementary Plate S16

**Plate S16 (preceding page).** Fossil Loranthaceae pollen from the late Oligocene/early Miocene Cottbus/Spremberg Formations, Germany. (A, D, I) LM micrographs. (B, C, E–H, J, K) SEM micrographs. (A–C) Altmittweida MT. (A) Polar view, convex-triangular grain, intercolpial nexine thickening at pole. (B) Polar view, syn(3)colpate grain with truncated equatorial apices. (C) Close-up showing sculpturing along margo and in area of mesocolpium. (D–H) Altmittweida MT. (D) Polar view, convex-triangular grain. (E) Polar view, syn(3)colpate grain with sexine partly reduced in polar area. (F) Close-up of equatorial apex. (G) Close-up of central polar area showing granulate colpus membrane. (H) Close-up showing sculpturing in area of mesocolpium. (I–K) Altmittweida MT. (I) Polar view, convex-triangular grain. (J) Polar view, syn(3)colpate grain. (K) Close-up of apex showing partly psilate to granulate margo and sculpturing in area of mesocolpium. Scale bars: (A, D, I) = 10 µm; (B, C, E–H, J, K) = 1 µm.
